# A Monoclonal Human Alveolar Epithelial Cell Line (“Arlo”) with Pronounced Barrier Function for Studying Drug Permeability and Viral Infections

**DOI:** 10.1002/advs.202207301

**Published:** 2023-02-07

**Authors:** Patrick Carius, Annemarie Jungmann, Marco Bechtel, Alexander Grißmer, Annette Boese, Gilles Gasparoni, Abdulrahman Salhab, Ralf Seipelt, Klaus Urbschat, Clémentine Richter, Carola Meier, Denisa Bojkova, Jindrich Cinatl, Jörn Walter, Nicole Schneider‐Daum, Claus‐Michael Lehr

**Affiliations:** ^1^ Helmholtz Institute for Pharmaceutical Research Saarland (HIPS) – Helmholtz Centre for Infection Research (HZI) Campus E8.1 66123 Saarbrücken Germany; ^2^ Department of Pharmacy Saarland University Campus E8.1 66123 Saarbrücken Germany; ^3^ Department of Genetics and Epigenetics Saarland University Campus A2 4 66123 Saarbrücken Germany; ^4^ Institute of Medical Virology University Hospital Frankfurt Paul‐Ehrlich‐Str. 40 60596 Frankfurt am Main Germany; ^5^ Department of Anatomy and Cellular Biology Saarland University Kirrberger Straße Building 61 66421 Homburg Saar Germany; ^6^ Section of Thoracic Surgery of the Saar Lung Center SHG Clinics Völklingen Richardstraße 5‐9 66333 Völklingen Germany

**Keywords:** drug transport, lung, pulmonary drug delivery, tight junctions, Transwell inserts

## Abstract

In the development of orally inhaled drug products preclinical animal models regularly fail to predict pharmacological as well as toxicological responses in humans. Models based on human cells and tissues are potential alternatives to animal experimentation allowing for the isolation of essential processes of human biology and making them accessible in vitro. Here, the generation of a novel monoclonal cell line “Arlo,” derived from the polyclonal human alveolar epithelium lentivirus immortalized cell line hAELVi via single‐cell printing, and its characterization as a model for the human alveolar epithelium as well as a building block for future complex in vitro models is described. “Arlo” is systematically compared in vitro to primary human alveolar epithelial cells (hAEpCs) as well as to the polyclonal hAELVi cell line. “Arlo” cells show enhanced barrier properties with high transepithelial electrical resistance (TEER) of ≈3000 Ω cm^2^ and a potential difference (PD) of ≈30 mV under air–liquid interface (ALI) conditions, that can be modulated. The cells grow in a polarized monolayer and express genes relevant to barrier integrity as well as homeostasis as is observed in hAEpCs. Successful productive infection with severe acute respiratory syndrome coronavirus 2 (SARS‐CoV‐2) in a proof‐of‐principle study offers an additional, attractive application of “Arlo” beyond biopharmaceutical experimentation.

## Graphical abstract

In vitro models based on human cells and tissues are a potential alternative to overcome the limitations of preclinical animal models, which regularly fail to predict the human response to inhaled drugs or noxae. The monoclonal human alveolar epithelial cell line “Arlo” reliably develops polarized monolayers with functional tight junctions, which inter alia allow for conducting drug transport and viral infection studies.

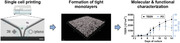

## 1 Introduction

The translational value of results from animal experiments for predicting the human response to orally inhaled compounds or drug products has been questioned in past years in inhalation research.^[^ ^1^, ^2^, ^3^ ^]^ Reasons include essential species‐species differences in anatomical structures as well as in physiological functions between different non‐human species and humans. For example, although genomic responses to inflammatory stimuli as observed in acute respiratory distress syndrome (ARDS) are highly similar within humans, these could not be reproduced using current mouse models.^[^ ^4^ ^]^ Complex in vitro models based on human cells and tissues were raised as a potential alternative to animal experimentation because they allow to reduce the complexity of human biology to an extent that can be consistently reproduced in vitro.^[^ ^5^ ^]^ The generation of such complex in vitro models, however, requires reliable human‐relevant cell sources as the essential building blocks, necessarily demonstrating reproducible experimental readouts in vitro.

In the case of the human lung, a morphologically and histologically complex organ, this means that different cell types prevailing in the diverse epithelial linings of the tracheobronchial region, the airways (bronchi) as well as the respiratory region (alveoli) of the peripheral lung would need to be considered.^[^ ^6^ ^]^ The highest number of different epithelial cell types exists in the approximately 50 µm thick epithelial lining of the bronchial airways. It is mainly characterized by a pseudostratified epithelium formed by columnar ciliated epithelial cells, club cells, mucus‐secreting goblet cells as well as basal cells that serve as progenitors to most of the airway epithelial cells.^[^ ^7^ ^]^ From proximal to distal, the surface that is covered by the epithelial lining of the human lung increases from around 4 m^2^ in the two bronchi to around 100 m^2^ spanning the alveolar epithelium.^[^ ^8^, ^9^ ^]^ This squamous epithelial layer has on average a delicate thickness of 1 µm and is formed by AT‐1 pneumocytes together with the cuboidal AT‐2 cells.^[^ ^10^ ^]^ While only representing about 8% of all lung cells, AT‐1 cells account for 95–98% of the total surface area of the alveolar epithelium due to a cell surface area of ≈5.000 µm^2^ per cell, ensuring effective gas exchange.^[^ ^8^, ^11^ ^]^ To prevent fluid leakage from the highly perfused vascular system underlying the alveolar epithelium into the air‐filled alveoli, a structure which is also called the air–blood barrier, the thin AT‐1 cells maintain tight cell‐to‐cell connections as well as effective fluid homeostasis by regulating water and ion transport. On the contrary, the cuboidal AT‐2 cells make up ≈18% of the cells of the alveolar epithelium but only contribute to 2–5% of the surface area, since they are mostly located at the corners of the alveoli.^[^ ^12^, ^13^ ^]^ AT‐2 cells produce pulmonary surfactant, a complex mixture of lipids and bioactive proteins, which prevents the alveolar sacs from collapsing by lowering the surface tension at the air–liquid interface above the epithelial lining.^[^ ^14^ ^]^ Furthermore, they contribute to the innate immune response and serve as facultative progenitors that differentiate into AT‐1 cells, but also replicate during epithelial homeostasis or after epithelial damage.^[^ ^15^ ^]^

When modeling pulmonary epithelia in vitro in the context of biopharmaceutical or toxicological inhalation experiments, air–liquid interface (ALI) conditions as well as tissue‐specific barrier integrity should be considered as standard requirements.^[^ ^16^, ^17^, ^18^ ^]^ For the bronchial epithelia described above, such cellular models are commercially available as primary cells from different donors supplied by various vendors or as continuous cell lines from recognized sources such as the American Type Culture Collection (ATCC) (extensively reviewed recently:^[^ ^19^, ^20^, ^21^ ^]^). In case of the alveolar epithelium though, cellular in vitro models that recapitulate tissue‐specific barrier integrity, provided by polarized alveolar epithelial cells that grow in a monolayer and show the formation of functional tight junctional complexes, were so far limited to primary human alveolar epithelial cells (hAEpCs) grown on porous growth supports (e.g., Transwell inserts)^[^ ^22^, ^23^, ^24^, ^25^ ^]^ or within advanced lung‐on‐chip devices.^[^ ^26^, ^27^, ^28^ ^]^

Freshly isolated primary hAEpCs seeded on Transwell inserts are still considered as a gold standard when replicating the human alveolar epithelium in vitro, especially during biopharmaceutical in vitro permeability experiments.^[^ ^20^ ^]^ Nevertheless, they come with several disadvantages such as costly isolation procedures, donor‐to‐donor variations or low yields that limit the experimental scale. Further, the isolated cells which are by the majority AT‐2 cells tend to transdifferentiate into AT‐1 cells when cultured on permeable growth supports, which is a wanted effect to reach proper barrier integrity, but then continue to further de‐differentiate resulting in a loss of barrier integrity and a limited experimental window of several days.^[^ ^29^, ^30^, ^31^, ^32^ ^]^ Impressive advances that were achieved by optimizing the differentiation protocols used to culture adult stem cells or human induced pluripotent stem cells and differentiate them further into organoids, yielded models that recapitulate the phenotypic as well as many other functional characteristics of the cells of the alveolar epithelium over an enhanced culture period.^[^ ^33^ ^]^ Whereas these models have the potential to overcome the limitations underlying the use of freshly isolated hAEpCs such as limited yield or availability in general, they currently face weak barrier properties,^[^ ^34^, ^35^, ^36^, ^37^ ^]^ inhomogeneous multiple cellular layers^[^ ^38^ ^]^ or multilayered closed alveospheres.^[^ ^39^, ^40^ ^]^

Continuously growing cell lines, derived from tumor biopsies or functionally immortalized by different genetic modifications, offer experimental convenience due to higher yields and flexible expansion, but might suffer from genetic instability as well as phenotypes distinct from epithelial cells in situ.^[^ ^20^, ^41^ ^]^ For example, the quite commonly used continuous cell line A549, originally derived from a human adenocarcinoma patient and showing some AT‐2‐like characteristics, lacks functional tight junctions, which disqualifies it for transport studies, at least as far as small molecules are concerned.^[^ ^42^ ^]^ Another report further demonstrated that the cell line is polyclonal and might yield up to three different sub‐clones.^[^ ^43^ ^]^

Recently though, two continuous cell lines, the adenocarcinoma‐derived NCI‐H441 as well as the human alveolar epithelial lentivirus immortalized (hAELVi) cell line, have been proposed as promising models for the alveolar epithelium especially in the context of biopharmaceutical experiments.^[^ ^44^ ^]^ While both cell lines present electrically tight epithelial layers with intercellular junctions forming a diffusion barrier as well as certain markers representative for alveolar epithelial cells, the hAELVi cell line maintains tight barrier properties under ALI conditions over a longer culture period than NCI‐H441.^[^ ^45^, ^46^, ^47^ ^]^

Unfortunately, the original hAELVi cell line presents a heterogeneous cell population due to the initial immortalization of a mixed population of CD326‐positive hAEpCs, without further monoclonal selection.^[^ ^45^, ^48^ ^]^ This led to inconsistencies in the development of barrier properties depending on the used cell culture vial, experienced by the authors of this study as well as by other laboratories leading to irreproducible experimental results.^[^ ^49^, ^50^, ^51^ ^]^

We here report the development as well as the characterization of a new monoclonal cell line named “Arlo.” The cell line is based on a single‐cell clone of the polyclonal hAELVi cell line generated by single‐cell printing and shows enhanced barrier properties while preserving a monolayer morphology. A transcriptomic analysis of “Arlo” in comparison to hAEpCs, both cultured under ALI conditions on Transwell inserts for a total of 14 days, demonstrated high similarity in gene expression relevant to barrier integrity and homeostasis. To show the versatility of “Arlo” beyond biopharmaceutical experimentation, we additionally report the infection of the new cell line with SARS‐CoV‐2 in a proof of principle study. In addition, the data from the transcriptomic analysis are publicly available as a resource for other researchers.

## 2 Results

### 2.1 Generation of the Single Cell Clone “Arlo”

The hAELVi cell line was generated based on the immortalization of a single‐donor hAEpC isolation of CD326‐positive (also known as an epithelial cell adhesion molecule (EpCAM)) cells that have been cultured on a six‐well cell culture plate for 5 d.^[^ ^45^, ^48^ ^]^ Functional immortalization was achieved by using self‐inactivating lentiviral vectors, comprising 33 genes under the control of a SV40 promotor.^[^ ^52^ ^]^ This procedure yielded two continuously growing polyclonal cell lines that were selected upon developing transepithelial electrical resistance (TEER) values >1000 Ω cm^2^ that were named hAELVi.A and hAELVi.B.

hAELVi.A was renamed to CI‐hAELVi and distributed by the company Inscreenex GmbH. CI‐hAELVi comprises a heterogeneous cell population that, depending on the initially thawed culture vial, would either demonstrate barrier formation indicated by TEER values >1000 Ω cm^2^ or would show impaired barrier formation (Figure S1, Supporting Information).

**Figure 1:**
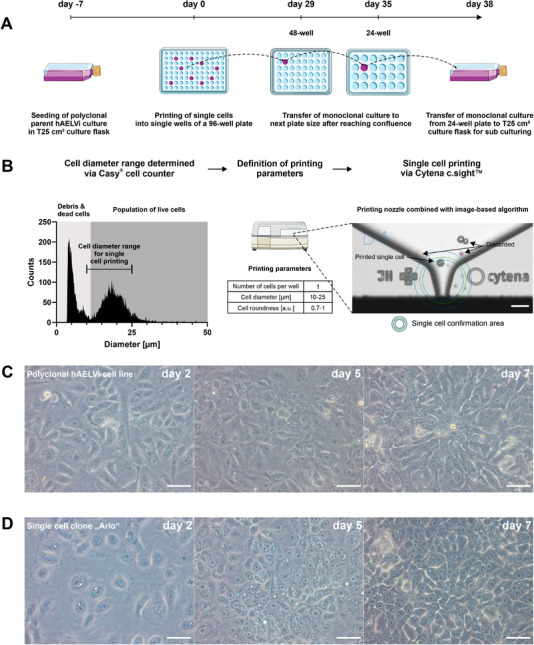
Generation of the single cell clone “Arlo.” A) Schematic depicting the single‐cell printing procedure (detailed in B) and subsequent passaging strategy for the single‐cell clone “Arlo” originating from a polyclonal hAELVi suspension that demonstrated TEER values >1000 Ω cm^2^ in the previous passage. B) Before single‐cell printing, the cell diameter of the polyclonal hAELVi cell suspension was determined via a Casy cell counter to define the printing parameters. Single cells within the printing parameters (bordered green) were confirmed by an image‐based algorithm and then deposited into a single well of a 96‐well plate. Cells that did not meet the printing criteria were discarded via vacuum aspiration after ejection from the printing nozzle (bordered purple or red). C,D) Light microscopic images showing morphological differences between C) the polyclonal hAELVi cell line and D) the single cell clone “Arlo” when cultured in T25 cm^2^ culture flasks for 7 d. Scale: 50 µm for all images displayed. Panel A) was partly generated using Servier Medical Art, provided by Servier, licensed under a Creative Commons Attribution 3.0 unported license.

In an initial effort to ensure reproducibility of experimental results from the hAELVi cell line, single‐cell clones were generated by single‐cell printing from a polyclonal hAELVi cell suspension that demonstrated barrier formation in the previous passage (**Figure** **1**A). The parameters needed for the single‐cell printing were determined via counting of the cell suspension with a Casy cell counter (Figure 1B). The image‐based algorithm of the Cytena c.sight single‐cell printer detected single cells and precisely deposited them within a drop via an inkjet‐like technique into a single well of a 96‐well cell culture plate. A series of pictures documents this process, and is exemplarily shown for well D4 that resembles the single cell clone “Arlo” (Figure S2, Supporting Information). After single‐cell printing, the single‐cell clone “Arlo” was serially expanded in multiple sized well‐formats until it eventually could be transferred into a T25 cm^2^ cell culture flask (Figure 1A). For reasons of consistency, we use the naming “single cell clone “Arlo”” whenever “Arlo” is compared to the polyclonal hAELVi cell line and the term “Arlo” in all other cases, including comparisons with hAEpCs. A visual comparison of the growth of the polyclonal hAELVi cell line (Figure 1C) and the single cell clone “Arlo” (Figure 1D) in T25 cm^2^ cell culture flasks already indicated a more homogeneous appearance in the case of the single cell clone “Arlo” in comparison to the polyclonal hAELVi cell line, especially on day 2 and day 5 of culture.

### 2.2 Electrophysiological and Functional Characterization of Barrier Properties of the Single Cell Clone “Arlo”

When epithelial cells are cultured on permeable growth supports, the increase in ohmic resistance of the in vitro tissue over time reported as TEER values serve as a non‐destructive, accepted measure to assess and monitor the formation of tight junctions as well as other cell‐to‐cell connections. When using traditional direct current based voltohmmeter such as the EVOM 2 to measure TEER values, epithelial potential difference (PD) can be measured as well using the same experimental setup. PD delivers additional information about the formation as well as homeostasis of an ion gradient by the epithelial cells in in vitro culture.

**Figure 2:**
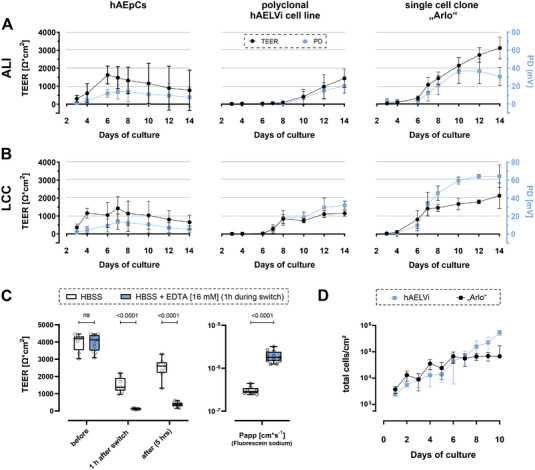
Electrophysiological and functional characterization of barrier properties of the single cell clone “Arlo.” A,B) TEER values (Ω cm^2^, black curve) as well as epithelial potential difference (PD) (mV, blue curve) of hAEpCs, the polyclonal hAELVi cell line and the single cell clone “Arlo” grown on Transwell inserts either under A) ALI or B) LCC conditions for 14 d. Data represent mean ± S.D. from 12 TWs and three independent biological replicates (hAEpCs day 4: 8 TWs; 2 bio. replicates). C) Apparent permeability of Fluorescein sodium (1 mg mL^−1^ in HBSS) transported over “Arlo” monolayers after 14 d of culture under LCC. TEER values were measured with cells cultured in the medium before the experiment (before), 1 h after the incubation in transport buffer (1 h after switch), as well as after the transport study (after). Transport buffer (HBSS) was supplemented with EDTA (16 × 10^−3^ m) during the 1 h incubation to disrupt tight junction complexes in one group. (TEER) 2‐way ANOVA was performed not assuming sphericity and with a Šídák´s multiple comparisons test. (*P*_app_) Unpaired *t*‐test was performed with Welch's Correction; Data represent mean ± S.D.; HBSS: *n* = 9, HBSS+EDTA: *n* = 12 from 3 independent biological replicates. D) Growth curve comparing the polyclonal hAELVi cell line with the single cell clone “Arlo”. Data represent mean ± S.D. from at least 2 technical replicates of 3 biological replicates (“Arlo”d10: single biological replicate).

In order to assess the capability of the single‐cell clone “Arlo” to develop and maintain an electrically tight barrier, we compared its barrier development against the polyclonal hAELVi cell line as well as hAEpCs over a course of 14 d either cultured under ALI (**Figure** **2**A) or under liquid‐covered conditions (LCC) (Figure 2B).

hAEpCs grown under ALI conditions showed an increase in TEER values from day 2 to day 6 (peak: 1624 ± 501 Ω cm^2^), then TEER values declined towards day 14 (772 ± 1122 Ω cm^2^). Under LCC, TEER values peaked at day 7 (1428 ± 656 Ω cm^2^) until they eventually declined to 654 ± 397 Ω cm^2^ on day 14. PD followed the same development as the TEER values, peaking at 14 ± 11 mV on day 8 under ALI conditions, as well as on day 7 at 13 ± 8 mV under LCC. Electrophysiological properties under both growth conditions were in accordance with our historic measurements.^[^ ^25^, ^29^ ^]^ The polyclonal hAELVi cell line showed a stepwise increase in TEER as well as PD development beginning from day 7 under LCC or day 10 under ALI conditions, respectively. TEER values reached a peak (ALI: 1436 ± 397 Ω cm^2^; LCC: 1148 ± 151 Ω cm^2^) on day 14, in a similar development as the PD values (ALI: 20 ± 7 mV; LCC: 31 ± 4 mV).

The single cell clone “Arlo” however, demonstrated a steadily increasing TEER development with TEER values about twice as high as the polyclonal hAELVi cell line on day 14 (ALI: 3112 ± 607 Ω cm^2^; LCC: 2136 ± 711 Ω cm^2^). A similar development could be observed for PD values, although these were appreciably higher under LCC (LCC: 64 ± 12 mV) than under ALI conditions (ALI: 37 ± 14 mV).

In order to assess whether the high TEER values developed by the single‐cell clone “Arlo” also correspond to the formation of functional tight junction complexes, we performed a transport experiment with the well‐defined low‐permeability marker fluorescein sodium (Figure 2C, left). One of the two experimental groups was treated with 2,2′,2″,2′″‐(ethane‐1,2‐diyldinitrilo) tetra acetic acid (EDTA) for 1 h during the equilibration with transport buffer. EDTA leads to the reversible opening of tight junction complexes by primarily chelating extracellular Ca^2+^. The resulting change in intracellular Ca^2+^ concentrations eventually activates Protein Kinase C which increases paracellular permeability.^[^ ^53^ ^]^ TEER values were measured to monitor barrier integrity before, 1 h after the switch to transport buffer, which was supplemented with 16 × 10^−3^ m EDTA in one group, as well as after the transport experiment. Before the transport experiment, where cells were cultured 14 d under LCC in cell culture medium, no significant differences in TEER values were observed between the two groups (3988 ± 512 Ω cm^2^ (HBSS) vs 3971 ± 473 Ω cm^2^ (HBSS + EDTA [16 × 10^−3^ m])). The switch to the transport buffer HBSS led to a strong decrease in TEER values after the 1 h incubation in case of the untreated group (1509 ± 420 Ω cm^2^ (HBSS)), but still remained above 1000 Ω cm^2^. In case of the group that was treated with EDTA, TEER values decreased significantly to 120 ± 31 Ω cm^2^ indicating a successful opening of tight junctional complexes. After 5 h of transport with only HBSS as a transport buffer in both groups, the TEER values of the control group still differed significantly from the group treated with EDTA during the 1 h incubation (2491 ± 586 Ω cm^2^ (HBSS) vs 379 ± 128 Ω cm^2^ (HBSS + EDTA [16 × 10^−3^ m])). This significant difference was also reflected in the Papp values for fluorescein sodium, which was significantly lower in the control group (3 × 10^−7^ ± 6 × 10^−8^ cm s^−1^ (HBSS)) in comparison to the group treated with EDTA (2 × 10^−6^ ± 6 × 10^−7^ cm s^−1^ (HBSS + EDTA [16 × 10^−3^ m])) (Figure 2C, right). This indicated that the paracellular permeability of the single‐cell clone “Arlo” could be experimentally modulated regardless of its strong barrier properties indicated by high TEER values.

To exclude that the higher TEER values in case of the single‐cell clone “Arlo” originated from a higher number of cells present in the culture and thereby generating a higher electrical resistance, we performed a growth curve to compare the growth of the single‐cell clone “Arlo” against the growth of the polyclonal hAELVi cell line. The data of the growth curve indicated that the cells of both cell lines equally proliferated until day 6 of the culture (Figure 2D). From day 6 onwards the polyclonal cell line hAELVi seemed to have further proliferated until day 10, whereas the total cell number of the single‐cell clone “Arlo” seems to enter an equilibrium. Interestingly, day 6 is also the day that marks the formation of TEER values for both cell lines under LCC (Figure 2B).

### 2.3 Morphological Comparison of the Single Cell Clone “Arlo” and the Polyclonal hAELVi Cell Line

In theory, the increase of the electrical resistance of an epithelial in vitro tissue to the net ion flux could be caused by cells growing in a monolayer, where the cells are developing tightly connected functional tight junctions as well as other cell‐to‐cell connections. Another possibility would be that cells are growing in multiple cellular layers, thereby increasing resistance to the net ion flux.

**Figure 3:**
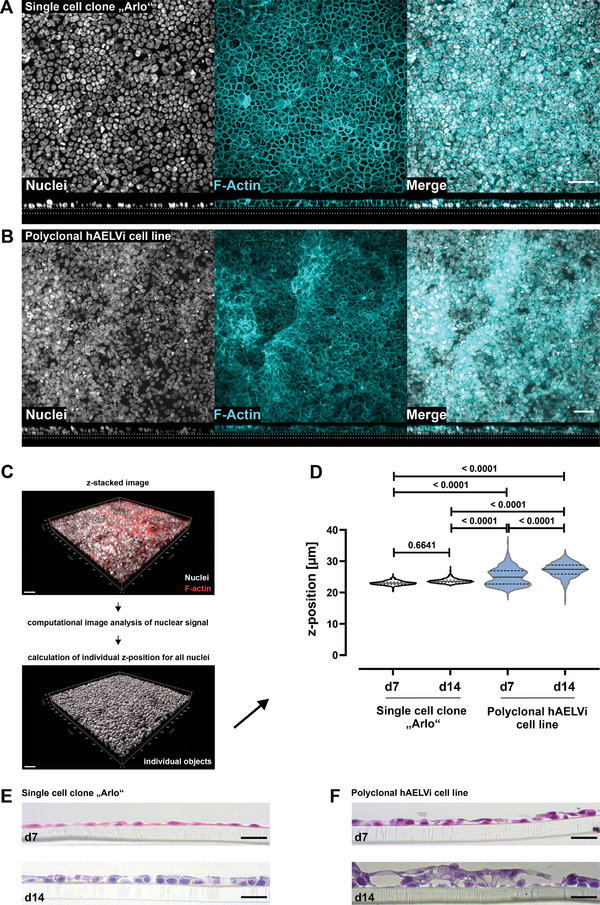
Different tissue morphology between the polyclonal hAELVi cell line and the single cell clone “Arlo.” A,B) Maximum projections from immunofluorescence staining showing cellular distribution as top view (upper panel) and the tissue morphology as orthogonal projections from z‐stacked images (lower panel) for A) the single cell clone “Arlo” as well as B) the polyclonal hAELVi cell line. Nuclei stained with DAPI (gray) as well as F‐Actin stained with phalloidin (cyan). Images are representative for 3 independent biological replicates. Scale bar: 50 µm. C) Schematic depicting the generation of individual digital objects for each cell, based on its nuclear signal by computational image analysis, computed from the information contained in z‐stacked confocal images. D) Computational image analysis of cellular z‐position computed from nuclear signal within different z‐stacks as a quantitative measure of the vertical cellular distribution within each in vitro tissue. 2‐way ANOVA was performed with a Tukey's multiple comparisons test. E,F) Histological tissue sections from the single cell clone “Arlo” as well as from the polyclonal hAELVi cell line stained with hematoxylin and eosin. Cells were grown under ALI conditions for 7 or 14 d. Representative for at least 2 biological replicates. Scale bar: 20 µm. A,B) For details concerning the deviating apparent scale bar please refer to the Experimental section.

To evaluate the tissue morphology of the single‐cell clone “Arlo” as well as the polyclonal hAELVi cell line, micrographs from confocal z‐stacks were analyzed (**Figure** **3**A,B).

Computed orthogonal sections within the horizontal plane of z‐stacked images, already indicated differences between the two cell lines in terms of layer morphology. In case of the single‐cell clone “Arlo” grown for 14 d under ALI conditions the top‐view micrograph as well as the orthogonal section of the representative z‐stacked image suggested that cells predominantly grew within a monolayer (Figure 3A). The polyclonal hAELVi cell line cultured under the same conditions as the single cell clone “Arlo” indicated the formation of multiple layers, as demonstrated by the top‐view micrograph as well as the orthogonal section of the z‐stacked image (Figure 3B).

Using only orthogonal image‐based sections of an in vitro tissue to assess the morphology of the cellular layer impairs the unbiased evaluation of the tissue. Sections need to be selected first and then assessed individually to deduce the layer morphology of the whole micrograph. In order to quantitatively assess the layer morphology of the single‐cell clone “Arlo” as well as the polyclonal hAELVi cell line, a computational image analysis was conducted (Figure 3C). For computational analysis, the signal of the nuclei from each cell was calculated as a single object within the 3D space of each z‐stack. The extracted z‐positions were then compared between the two cell lines (Figure 3D). The comparison of the distribution of the nuclear signal as a surrogate for the cellular position on the z‐axis in relation to the Transwell‐membrane within each layer showed, that the single cell clone “Arlo” grew in a monolayer on day 7 of culture under ALI conditions and also displayed a monolayer morphology until day 14 of culture. The polyclonal cell line hAELVi however, showed the formation of multiple layers already at day 7 of culture under ALI conditions. This was also observed on day 14, differing significantly from the single‐cell clone “Arlo” as well as from the condition on day 7.

Representative histological sections of the in vitro tissue further supported the results of the computational image analysis, which was based on immunofluorescence staining, by another method. The single cell clone “Arlo” maintained a monolayer morphology over the 14 days of culture under ALI conditions, whereas the polyclonal cell line hAELVi seemed to develop multiple layers already on day 7 of culture under ALI conditions and even more pronounced on day 14 (Figure 3D).

### 2.4 Similarities in Gene Expression Relevant to Barrier Integrity between hAEpCs and “Arlo”

Notably enough, the newly created “Arlo” cells demonstrated remarkably high TEER values, experimentally adaptable tight junction complexes as well as stringent monolayer morphology. To assess whether “Arlo” could be used as a model for the alveolar epithelium, not only on the functional but also on the molecular level, a bulk RNA‐Sequencing analysis (RNA‐seq) was conducted. The RNA‐seq results from “Arlo” were compared to results from hAEpCs both cultured under the same conditions for 14 days under ALI. Samples for RNA‐seq have been collected before seeding cells on Transwell inserts (day 0), as well as on day 7 and day 14 of culture. The day 0 samples from the hAEpCs used for RNA‐seq were generated from the CD326/EpCAM‐positive cellular fraction of the donor tissue on the day the tissue was resected and thus stand representative for the RNA expression status of the epithelial fraction of the native tissue. The day 0 samples from “Arlo” used for RNA‐seq were generated from freshly passaged cells, which were continuously cultured in T25 cm^2^ culture flasks for 7 d prior to passaging.

**Figure 4:**
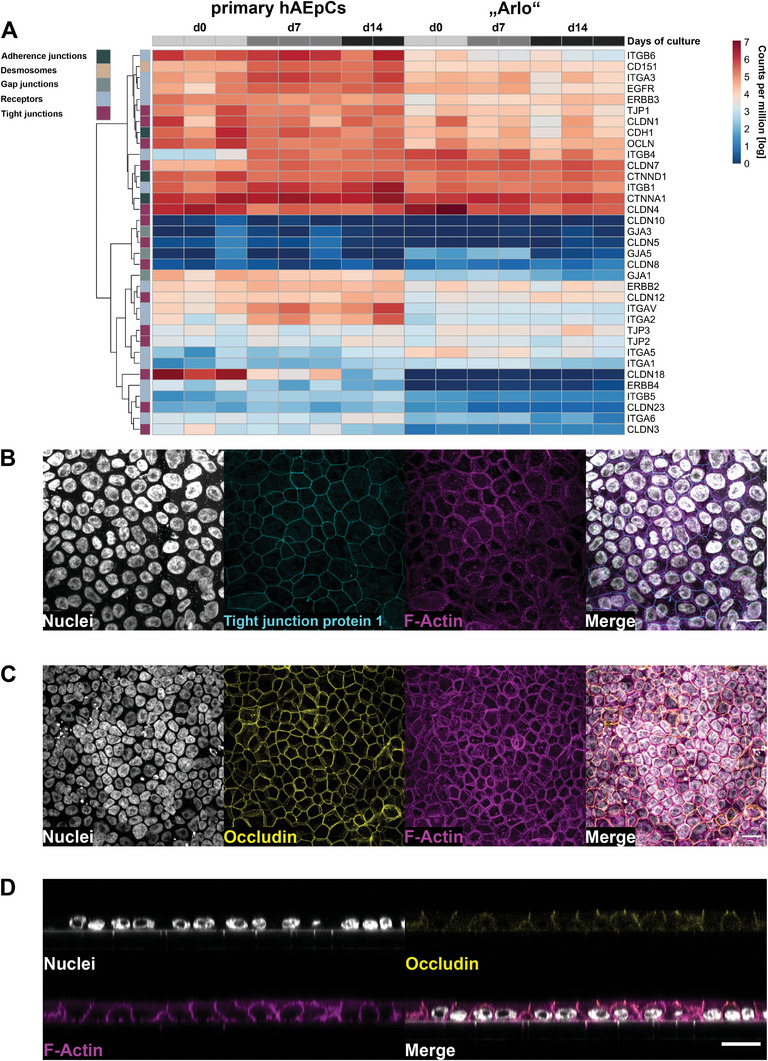
Similar expression of barrier relevant genes by “Arlo” in comparison to hAEpCs. A) Expression of 35 genes associated with regulating lung barrier integrity or barrier homeostasis by hAEpCs and the single cell clone “Arlo” cultured under ALI conditions was determined via bulk RNA‐Sequencing. Genes were assigned the following classes: adherence junctions, desmosomes, gap junctions, receptors and tight junctions based on the function of their respective gene products. Data represent at least 2 biological replicates. B‐C) Representative confocal maximum projections from “Arlo” cells on day 14 of culture under ALI conditions, demonstrating homogenously connected networks of the barrier related proteins tight junction protein 1 (B, TJP1, cyan) and Occludin (C, yellow) stained by immunofluorescence. D) Orthogonal projection from confocal microscopy indicating an apically located Occludin signal. Nuclei stained with DAPI (gray) as well as F‐Actin stained with phalloidin (magenta) were included as structural controls in all micrographs. Scale bar: 20 µm.

First, the expression of 35 genes whose products are associated with regulating lung barrier integrity or barrier homeostasis were identified in the literature and compared between hAEpCs as well as “Arlo” (**Figure** **4**A).^[^ ^54^, ^55^, ^56^ ^]^

The genes were assigned to the following classes: adherence junctions, desmosomes, gap junctions, receptors, and tight junctions based on the associated function of their gene products. The tight junction class of gene products directly regulates the paracellular permeability of ions and hydrophilic small molecules, specifically through adhesive transmembrane proteins (e.g., Claudins). However, gene classes whose products contribute to structural epithelial integrity (e.g., adherence junctions) as well as cellular communication (e.g., gap junctions) were also included in this comparison to additionally cover other important epithelial functions. The striking majority of the 35 genes showed similar expression levels in both hAEpCs as well as “Arlo,” as indicated by 4 broader categorized apparent clusters. Clearly distinguishable differences in gene expression were observed within the receptor class. The genes coding for the integrin subunits ITGA2, ITGA5, ITGAV, and to a lesser extent the genes ITGA6, ITGB4 as well as ITGB6 demonstrated differing expression levels either in general or at specific time points between the two cellular models. Further, the gene CLDN18 that codes for the alveolar‐relevant tight junctional protein claudin‐18 did not show a detectable expression in “Arlo” whereas its expression declined in hAEpCs from day 0 to day 14 of culture under ALI conditions on Transwell inserts. In addition, the gene ERBB4 (HER4), that codes for the disease‐relevant Erb‐b2 receptor tyrosine kinase 4, could also not be detected within “Arlo” whereas a moderate expression was observed within hAEpCs.

From these 35 genes, the resulting structural junctional proteins Occludin as well as tight junction protein 1 (ZO‐1) were chosen as empirically‐defined molecules representative for functional tight junction complexes mainly located apically in polarized epithelial cells.^[^ ^57^, ^58^ ^]^ The elevated gene expression of the respective genes OCLN (Occludin) as well as TJP1 (Tight junction protein 1) observed for “Arlo” already indicated that the respective proteins might also be detectable (Figure 4A–C). Micrographs of fluorescent immunocytochemistry staining showed a homogenously distributed and continuously connected network of Tight junction protein 1 (Figure 4B) as well as an Occludin signal (Figure 4C). Orthogonal optical sections obtained from confocal microscopy additionally showed an overlap of Occludin and F‐Actin signal at the apical cellular junction, further indicating that “Arlo” demonstrates physiologically relevant cellular polarization. In addition, the same data also again showed the monolayer morphology displayed by “Arlo.”

**Figure 5:**
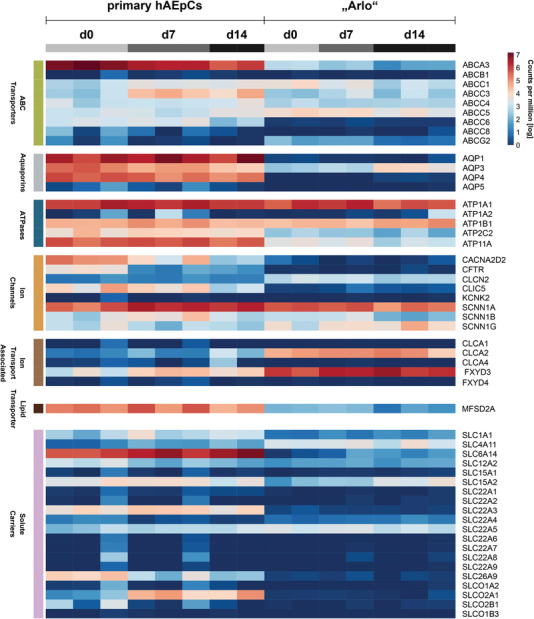
Expression of genes related to molecular and ion transport by “Arlo." Genes were assigned to the following classes: ABC transporters, aquaporins, ATPases, ion channels, ion transport associated, lipid transporter and solute carriers based on the function of their respective gene products. Genes showed expression in the human lung and were selected from literature.^[^ ^56^, ^59^, ^60^, ^61^, ^62^ ^]^ Data represent at least 2 biological replicates of cells cultured under ALI conditions and were derived from bulk RNA‐sequencing.

The ability of “Arlo” to form a polarized epithelium with functional tight junctions is also the basis for the establishment of vectorial ion transport. “Arlo” already demonstrated elevated PD values under ALI conditions (Figure 2A, right; ALI: 37 ± 14 mV) when compared to the polyclonal hAELVi cell line as well as to hAEpCs. To assess whether the elevated PD values are related to an elevated synthesis of proteins involved in molecular transport, the expression of 52 genes whose products are involved in the active or passive transport of ions, small‐ and macromolecules as well as drugs was assessed (**Figure** **5**).

The selected genes were shown to be expressed in the human lung and were derived from literature.^[^ ^56^, ^59^, ^60^, ^61^, ^62^ ^]^ The following classes categorize the genes based on the function of their respective gene products: ABC transporters, aquaporins, ATPases, ion channels, ion transport associated, lipid transporter and solute carrier. Gene expression was compared between “Arlo” and hAEpCs over 14 d of culture under ALI conditions. The expression level of many genes was comparable between samples of “Arlo” and hAEpCs, including the genes coding for the sodium/potassium‐transporting ATPase catalytic (*α*) and regulatory (*β*) subunits (ATP1A1, ATP1A2, ATP1B1) or the sodium channel epithelial 1 subunits (also known as ENaC) subunits (SCNN1A, SCNN1B) within the ATPases class. Among the genes with comparable expression were also the ones coding for drug transporters: ABCB1 (also known as MDR1 or P‐gp), ABCC1 (MRP1), ABCC4 (MRP4), ABCC5 (MRP5), ABCG2 (BCRP), SLC15A2 (PEPT2) or SLC22A5 (OCTN2). The gene coding for the sodium channel epithelial 1 subunit gamma (SCNN1G), showed a slight increase in expression within samples of “Arlo” during the course of ALI culture. The expression of the CFTR gene, which codes for the cystic fibrosis transmembrane conductance regulator, seems to be absent in “Arlo,” while CFTR expression was well present in hAEpCs, especially in day 0 samples.

Two genes of the ion transport associated class, however, FXYD3 and CLCA2, showed an elevated gene expression within “Arlo” samples. The respective protein (FXYD domain containing ion transport regulator 3), which the FXYD3 gene codes for, regulates the activity of the sodium/potassium‐transporting ATPase.^[^ ^63^ ^]^ The gene product of the CLCA2 gene, the Chloride channel accessory 2 protein modulates calcium‐activated chloride channel currents.^[^ ^64^ ^]^ Further, the genes ABCA3 within the ABC transporter class, the genes AQP1, AQP3, AQP4 within the aquaporin class, ATP11A within the ATPases class, MFSD2A within the lipid transporter class and SLC6A14 within the solute carrier class showed an elevated expression within hAEpCs samples.

### 2.5 Cellular Identity of “Arlo”

The polyclonal hAELVi cell line was considered to have an AT‐1‐like character.^[^ ^45^ ^]^ Given that the cell line demonstrated elevated TEER values under different culture conditions, the AT‐1‐like assumption was in parts based on the observed elevated expression of CAV1 as well as on a nondetectable SFTPC expression. The expression of CAV1, the gene coding for the protein Caveolin 1, is enhanced in ATI pneumocytes and SFTPC expression, the gene coding for Surfactant protein C, is considered a specific molecular marker for AT‐2 pneumocytes.^[^ ^65^, ^66^ ^]^

In an effort to determine cell type‐specific gene signatures present within the hAEpCs in vitro cultures as well as in the cultures of “Arlo,” the bulk RNA‐seq data generated in this study was mapped against the most current set of consensus marker genes for epithelial cells in the human lung.^[^ ^59^ ^]^ The marker genes provided by the integrated Human Lung Cell Atlas define a set of genes that in their specific combination show an elevated expression within the different cell types of the human lung and are used to identify the respective cell types. The data underlying the Human Lung Cell Atlas are consensus‐based and represent the most recent re‐annotation of 46 single‐cell RNA‐seq data sets of the human respiratory system.

**Figure 6:**
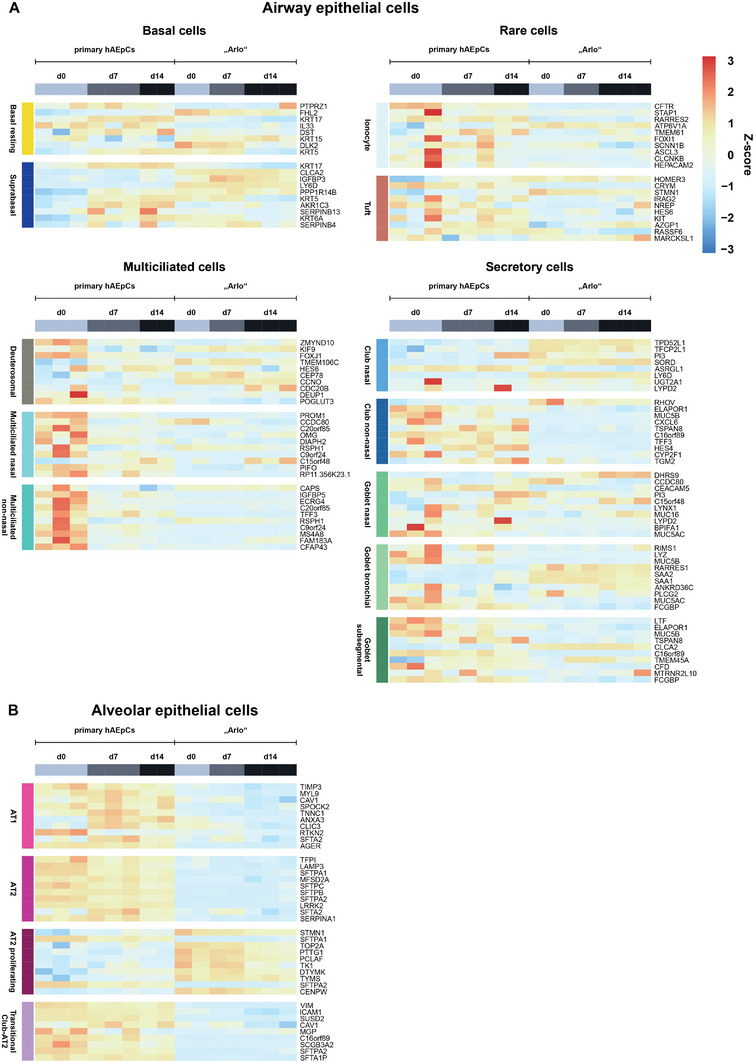
Cell type specific gene signatures in the samples of hAEpCs and “Arlo.” Cell type specific gene signatures of genes whose expression is representative for specific epithelial cell types within the human lung were defined by and derived from the integrated Human Lung Cell Atlas consortium.^[^ ^59^ ^]^ A) Airway epithelial cell types are subdivided into basal cells, multiciliated cells, rare cells and secretory cells. Individual cell types are displayed on each heatmap (left, vertically). B) Alveolar epithelial cells are categorized into individual cell types without further sub‐division. Data represent at least 2 biological replicates of cells cultured under ALI conditions and were derived from bulk RNA‐Sequencing.

The data presented here is broadly categorized into the analysis of airway epithelial cells (**Figure** **6**A) and alveolar epithelial cells (Figure 6B). The airway epithelial cell analysis covers basal cells (subdivided into: basal resting, suprabasal), multiciliated cells (subdivided into: deuterosomal, multiciliated nasal, multiciliated non‐nasal), secretory cells (sub‐divided into: club nasal, club non‐nasal, goblet nasal, goblet bronchial, goblet subsegmental) and rare cells (sub‐divided into: ionocyte, Tuft). The analysis of the alveolar epithelial cells covers AT‐1 cells, AT‐2 cells, AT‐2 proliferating cells as well as transitional club‐AT‐2 cells.

The analysis showed that in case of the hAEpCs, on the day of the isolation (day 0) many transcripts which are related to airway epithelial cells (especially multiciliated nasal, multiciliated non‐nasal and club non‐nasal) are overrepresented indicating a presence of these cell types within the hAEpCs on day 0 from all donors. Interestingly, in one donor isolation the transcripts representative for rare ionocytes and tuft cells seem to be overrepresented (Figure 6A). It stands out however, that hAEpCs in general, but especially on day 7 as well as day 14 of culture, show a distinct overrepresentation of most transcripts representative for alveolar epithelial cells (AT‐1 cells, AT‐2 cells, and transitional club‐AT‐2 cells) (Figure 6B).

Interestingly enough, in case of “Arlo” most of the overrepresented gene signatures could be referred to proliferating AT‐2 cells. Although CAV1 as well as a few other AT‐1 cell related genes (ANXA3, CLIC3 and SFTA2) seem to be overrepresented on day 0 and day 7 in the samples of “Arlo,” the majority of mature AT‐1 as well as AT‐2 related genes seemed to be underrepresented. The data further indicate that gene signatures of suprabasal as well as club cells from nasal origin are overrepresented within “Arlo” (Figure 6A).

In sum, the gene signatures present in “Arlo” seem to be representative of different cell types, but primarily match proliferating AT‐2 cells. Gene signatures found in “Arlo” stand in contrast to the gene signatures observed in hAEpCs, which mostly represent AT‐1 cells, AT‐2 cells, and transitional club‐AT‐2 cells.

**Figure 7:**
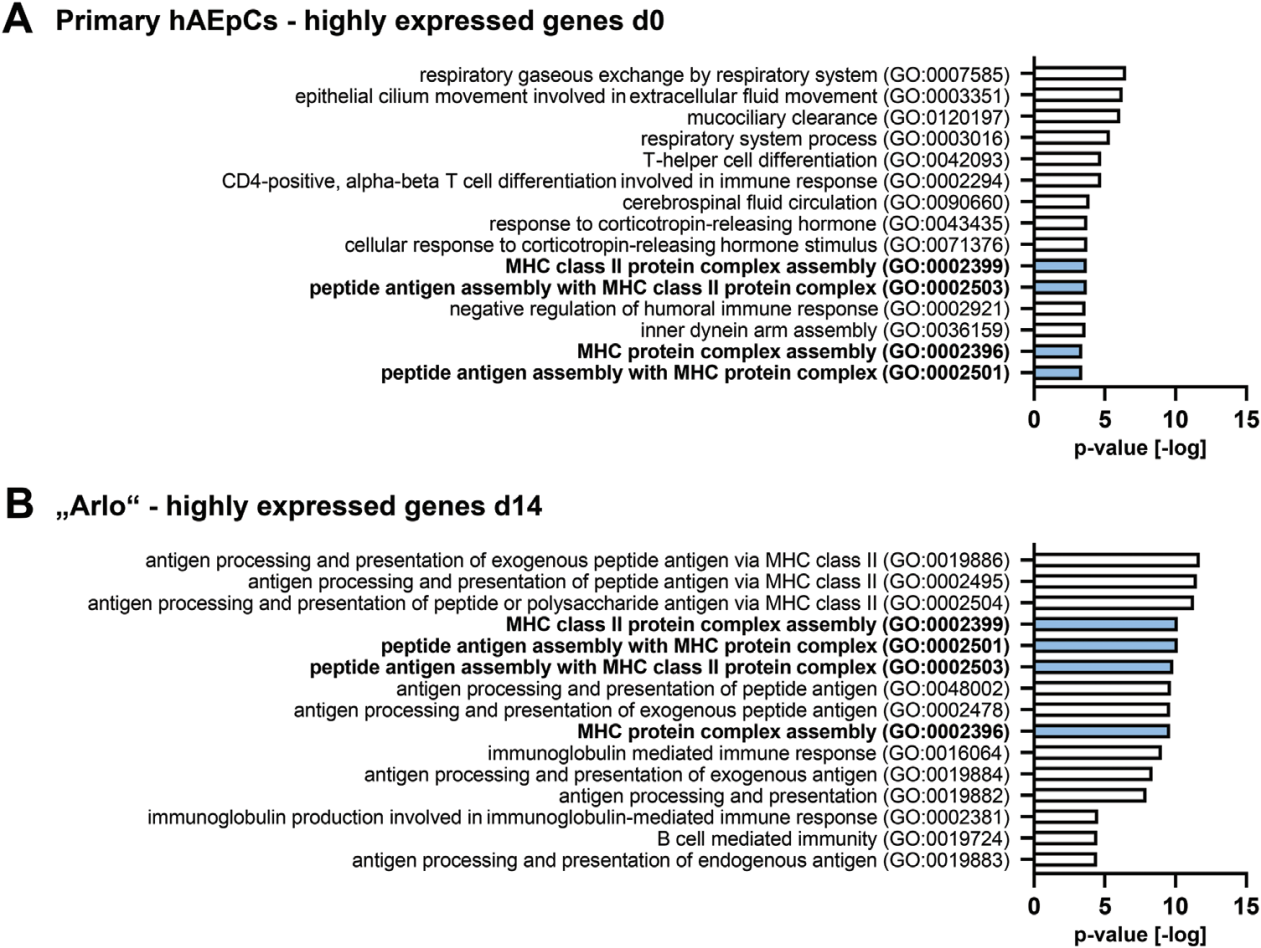
Gene ontology analysis reveals expression of MHC II surface antigens by hAEpCs and “Arlo.” Gene ontology (GO) analysis of the top 15 highly expressed genes in hAEpCs on A) day 0 after isolation and in “Arlo” on B) day 14 of culture under ALI conditions.^[^ ^69^, ^70^, ^71^, ^72^, ^73^ ^]^ Shared gene ontology terms are marked in blue. Data represent at least 2 biological replicates of cells cultured under ALI conditions and were derived from bulk RNA‐sequencing.

In support of these findings, a gene ontology analysis demonstrated that hAEpCs on day 0 (**Figure** **7**A) of culture and “Arlo” on day 14 of culture (Figure 7B) share gene ontology terms that relate to the expression of surface antigens of the major histocompatibility complex (MHC) II. Expression of these surface antigens was shown for AT‐2 cells.^[^ ^67^, ^68^ ^]^

These findings were further supported when traditional, empirically derived markers for alveolar epithelial cells were analyzed (Figure S3, Supporting Information).^[^ ^15^ ^]^ In the case of “Arlo,” some elevated transcripts of the AT‐1‐like marker CAV1 could be observed on day 0 and day 7, together with some elevation in the transcripts of the AT‐2‐like markers ABCA3, LAMP3 and MUC1 especially on day 0. These were however lower in abundance when compared to hAEpCs. The basal cell marker KRT15, and the secretory club cell marker TUBB3 were the only transcripts from the empirical marker set which displayed higher abundance in “Arlo” than in hAEpCs.

### 2.6 “Arlo” as a Use‐Case to Study Viral Infections of the Deep Lung

**Figure 8:**
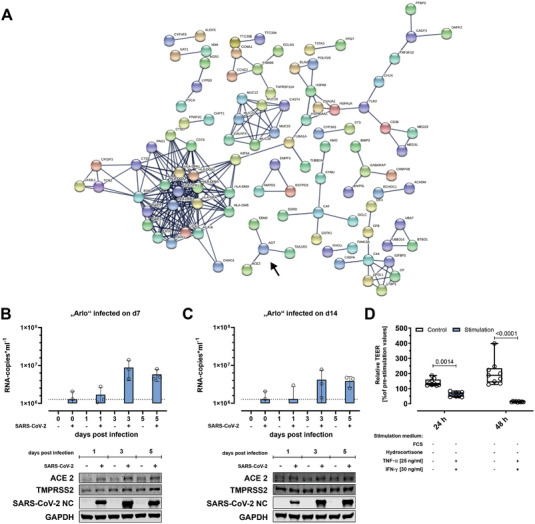
SARS‐CoV‐2/FFM7 infection as use‐case for using “Arlo” for viral infection studies. A) Cluster analysis by the STRING resource to obtain potential protein‐protein interactions present in samples of the single‐cell clone “Arlo.” Arrow marks a cluster around the gene AGT that indicates an interaction with the gene ACE2. Analysis was derived from bulk RNA‐Sequencing data of cells cultured until day 14 under ALI conditions. Data represent at least 2 biological replicates. B,C) Infection studies performed with “Arlo” either on A) day 7 or B) day 14 of culture under ALI conditions with SARS‐CoV‐2/FFM7 (MOI of 1) or mock (PBS). Upper panels show SARS‐CoV‐2/FFM7 RNA copy numbers (RNA‐copies mL^−1^) derived from qRT‐PCR of the RNA‐dependent RNA polymerase (RdRp) gene copies present in single apical washes (30 min, PBS) on the given days post‐infection. Lower panels show western blots indicating the cellular presence of angiotensin I converting enzyme 2 (ACE2), transmembrane serine protease 2 (TMPRSS2) and SARS‐CoV‐2/FFM7 nucleocapsid protein (SARS‐CoV‐2 NC) in samples infected with SARS‐CoV‐2/FFM7 (MOI of 1) or mock (PBS). Glyceraldehyde‐3‐phosphate dehydrogenase (GAPDH) was used as a control protein. Data represent single independent experiments. D) Reduction of barrier properties by stimulating “Arlo” monolayers synergistically with TNF‐*α* [25 ng mL^−1^] and INF‐*γ* [30 ng mL^−1^] for 48 h. 24 h before the experiment, cells were incubated in stimulation medium without FCS and hydrocortisone as an adaptation of a previous protocol.^[^ ^75^ ^]^ “Arlo” monolayers were grown under LCC for 10 d before the switch to stimulation medium. TEER values were normalized to the values before stimulation. Data represent mean ± S.D.; *n* = 9 for each group from 3 independent biological replicates.

The RNA expression data generated in this study were used to identify potential protein–protein association networks active in “Arlo.” To do so, the STRING resource, a comprehensive online database to discover protein–protein association networks from genome‐wide gene expression datasets, was harnessed.^[^ ^69^, ^70^ ^]^ From the several clusters of potential protein–protein interactions obtained from the “Arlo” dataset at day 14 under ALI conditions, the cluster around the gene AGT, which indicated functional interactions also among EDN2, TAS1R3 as well as ACE2 was identified as basis for further studies in the context of infection research (**Figure** **8**A). The pathogenic SARS‐CoV‐2 that first emerged in China in December 2019 and whose international pandemic spread led to a global health emergency, uses the angiotensin I converting enzyme 2, the protein encoded by ACE2, as an entry receptor for uptake by the host followed by the intracellular priming of the SARS‐CoV‐2 S protein by the serine protease TMPRSS2.^[^ ^74^ ^]^ In this context, SARS‐CoV‐2 infection experiments with “Arlo” were conducted as single biological experiments in a proof of principle study to determine the best time points for infection and to evaluate whether productive infection could be observed (Figure 8B,C). When “Arlo” was infected on day 7 of ALI culture, SARS‐CoV‐2 RNA‐copies progressively increased until day 3 postinfection and remained elevated until day 5 postinfection (Figure 8B, upper panel). Productive infection was further supported by western blot, were SARS‐CoV‐2 nucleocapsid protein was detected in parallel to the rising RNA‐copies (Figure 8B, lower panel). Infection of “Arlo” with SARS‐CoV‐2 on day 14 of ALI culture, also led to an increase in SARS‐CoV‐2 RNA‐copies, accompanied by evidence of productive infection indicated by nucleocapsid protein synthesis from day 3 postinfection and onwards. The total number of RNA‐copies, however, appeared to be reduced in comparison to the infection on day 7 (Figure 8C). Presence of the angiotensin I converting enzyme 2 as well as the serine protease TMPRSS2 was additionally confirmed in both experiments on the protein level (Figure 8B,C).

In patients that suffer from the coronavirus disease 2019 (COVID‐19), the disease caused by a SARS‐CoV‐2 infection, a synergism of the cytokines TNF‐*α* and INF‐*γ* triggers inflammatory cell death as well as tissue damage.^[^ ^76^ ^]^ Based on the adaptation of an existing protocol where cells are cultured in absence of FCS and hydrocortisone as stimulation medium, TNF‐*α* and INF‐*γ* stimulation was used to demonstrate cytokine induced reduction of barrier properties in case of “Arlo” (Figure 8D).^[^ ^75^ ^]^ Synergistic stimulation with TNF‐*α* and INF‐*γ* led to a relative decrease of ≈50% in TEER after 24 h compared to the TEER values before stimulation. After 48 h of stimulation with TNF‐*α* and INF‐*γ* a significant reduction of TEER values was observed.

The findings from the single‐variant infection studies described above were also used to infect “Arlo” with other variants of SARS‐CoV‐2 (FFM1, FFM 7, Alpha, Beta and Zeta) (Figure S4, Supporting Information). Although infection was not achieved for all of the variants, productive infection could be observed for some of them. Although investigation that is more thorough will be needed here, preliminary experiments suggest differences in barrier disruption responses to infection by the variants.

## 3 Discussion

We generated the single‐cell clone “Arlo” in light of the growing demand for a reproducible cellular in vitro model of the alveolar epithelium for biopharmaceutical inhalation research. “Arlo” not only demonstrates robust barrier integrity with TEER values >3000 Ω cm^2^ under ALI conditions, but in addition also develops a polarized monolayer. These functional barrier properties are further supported on the molecular level by the expression of genes and proteins related to barrier integrity as well as homeostasis that largely correlates with hAEpCs cultured in vitro.

To be used in biopharmaceutical inhalation experiments, such tissue‐specific barrier integrity provided by alveolar epithelial cells grown at the ALI could in the past almost exclusively be obtained in vitro by isolated primary hAEpCs that were differentiated towards AT‐1‐like cells on Transwell inserts.^[^ ^20^, ^23^, ^25^, ^29^ ^]^ Primary cell isolations are costly and the trans‐differentiation of these cells in vitro restricts their experimental use to only a few days. Sub‐cultivation of hAEpCs without the addition of feeder cells is possible for a very limited number of passages, but is mostly avoided due to rapid de‐differentiation of the cells which might influence experimental readouts.^[^ ^30^ ^]^

The rapid advancement of culture models that are either generated from human adult stem cells or induced pluripotent stem cells (IPSCs) that are often further differentiated into organoids could offer an alternative to the use of freshly isolated hAEpCs when modeling the alveolar epithelium in vitro.^[^ ^33^ ^]^ Due to their intact stemness during in vitro culture, these cells can be continuously expanded. When cultured under ALI conditions on Transwell inserts or on basement membrane equivalents some models demonstrated AT‐1 as well as AT‐2 marker expression such as aquaporin 5 (AQP5), podoplanin (PDPN) or SFTPC after differentiation.^[^ ^35^, ^36^, ^37^, ^39^ ^]^ Another model which is based on alveospheres also showed enhanced expression of markers relevant for alveolar epithelial cells, but is not suitable for standardized drug transport studies over the alveolar epithelium yet because the closed spheres are fully embedded in 3D‐culture matrices.^[^ ^40^ ^]^ Other models were either not cultured under ALI conditions or demonstrated the formation of inhomogeneous cellular multilayers when seeded on Transwell inserts.^[^ ^38^, ^77^ ^]^ What all of the aforementioned studies have in common, is that they either did not assess barrier integrity at all or reported TEER values <300 Ω cm^2^ and/or have not been characterized in terms of drug transport. In a model reported by He et al., human IPSCs were differentiated to alveolar organoids and in parallel to endothelial cells before both cell types were seeded on artificial or reconstituted basement membranes under ALI‐conditions. In this study, TEER values of ≈400 Ω cm^2^ were reported, but drug transport was not assessed.^[^ ^34^ ^]^ A further aggravating factor is that such models are experimentally challenging to establish, costly to implement and might hamper inter‐laboratory comparison due to complex differentiation protocols.^[^ ^77^, ^78^ ^]^ One alveolar epithelial model which is based on differentiated human IPSCs grown on 96‐well Transwell inserts under ALI conditions, reported tight barrier properties indicated by TEER values > 1000 Ω cm^2^ and would thus be suitable for drug transport studies.^[^ ^79^ ^]^ These TEER values, however, were measured using a custom‐built device and were only compared to literature derived TEER values from hAEpCs. Given the inter‐laboratory variability of TEER measurements in general, side‐by‐side comparisons using the same experimental setup should be performed when assessing TEER values, as previously discussed and also conducted in the current study.^[^ ^80^ ^]^ The provided confocal micrographs further indicated multilayered cellular clusters, which were not assessed quantitatively and could have influenced TEER development.

Although regarded as a promising in vitro model for the alveolar epithelium based on a continuous cell line, the polyclonal hAELVi cell line generated inconsistent results in the development of functional barrier properties as reported in the literature, most probably due to its polyclonality. The results reported in the current study for the polyclonal hAELVi cell line as well as experiments performed by other laboratories show that barrier properties developed differently over time than originally reported by Kuehn et al.^[^ ^49^, ^50^, ^51^ ^]^ The reason for these conflicting reports most probably is that the polyclonal hAELVi cell line was generated by functional immortalization of multiple CD326/EpCAM‐positive hAEpCs that have been cultured on a six‐well cell culture plate for 5 days without clonal selection.^[^ ^45^, ^48^ ^]^ When isolating alveolar epithelial cells only via CD326/EpCAM as a positive molecular selection marker, not only AT‐2‐like cells are selected from a cell suspension but also AT‐1‐like as well as bronchiolar epithelial cells.^[^ ^81^ ^]^ These findings are further supported by the transcriptomic analysis in this study, where gene signatures of these different epithelial cell types could also be detected in the hAEpCs on d0 after isolation, which have been isolated according to the same experimental protocol as the hAEpCs used for the generation of the polyclonal hAELVi cell line. Also in accordance with this hypothesis, other laboratories reported AT‐2‐like properties for a sub‐population of the polyclonal hAELVi cell line, such as formation of microvilli or presence of surfactant as well as of proteins related to surfactant secretion.^[^ ^82^, ^83^ ^]^ It needs to be mentioned though, that propagation of sub‐populations of the polyclonal hAELVi cell line could also be influenced by different growth media used to culture the polyclonal hAELVi cell line. We used equal culture conditions (cell culture medium and Transwell inserts) compared to the ones used by Kuehn et al. during the culture of all cellular models in the current study, whereas other reports used different growth media. Out of this reason, we highly recommend to follow the methods as well as to use the same culture media etc. described in this study, to ensure interlaboratory comparison of experimental results obtained from the single cell clone “Arlo” but also from the polyclonal cell line hAELVi.

The gene signatures representative for lung epithelial cells used in our study to assess the proportional cell types present in the primary hAEpCs isolations as well as in the “Arlo” samples were derived from the data published in the context of the human lung cell atlas.^[^ ^6^, ^59^ ^]^ To our knowledge, the data from the human lung cell atlas contain the most recent consensus‐based as well as the largest set of annotations of the different cell types within the human lung and were thus selected for the gene signature analysis. Based on this data, the transcripts identified in “Arlo” seem to resemble proliferating AT‐2 cells the most, followed by less abundant gene signatures representative for suprabasal as well as club cells of nasal origin. Apart from the abundance of gene signatures representative for proliferating AT‐2 cells, we additionally identified the expression of transcripts related to the synthesis of MHC II in the samples of “Arlo,” as verified by the gene ontology as well as STRING network analysis. Surface antigens such as the MHC II molecules HLA‐DR, HLA‐DP, or HLA‐DQ have been shown to be constitutively expressed by human AT‐2 cells where they act as a major contributor to barrier immunity in vivo and are being discussed as exclusive selection markers to obtain pure AT‐2 cell isolations.^[^ ^67^, ^68^, ^84^, ^85^ ^]^ Our gene ontology analysis data further indicate, that the expression of MHC II related molecules seems to be also highest in the hAEpCs samples immediately after isolation, before they decline over the course of in vitro culture. In the samples of “Arlo,” a contrary development was observed characterized by a high presence of transcripts related to antigen presentation up to day 14 of culture.

In general, “Arlo” seems to show a marker expression profile that is distinct from the gene signatures obtained from hAEpCs. In the context of a potential AT‐2‐like phenotype of “Arlo,” this is most pronounced in the expression levels of surfactant related transcripts. Transcripts for SFTPA1, SFTPA2, SFTPB, SFTPC as well as SFTPD show no to very low expression in “Arlo.” Although SFTPC expression is considered the current gold standard to identify mature AT‐2 cells, recent data suggest a heterogeneity among the adult AT‐2 population in vivo.^[^ ^6^ ^]^ In this context, AT‐2 cells that show a low to absent SFTPC expression but a higher expression of ABCA3 seem to represent a proliferating progenitor cell type, while AT‐2 cells that show high SFTPC levels together with a high expression of ABCA3 seem to rather represent mature AT‐2 cells.^[^ ^86^ ^]^ The authors speculated that these progenitor populations might be activated during early lung development, during disease or after lung damage. ABCA3 expression, however, declines in “Arlo” from day 0 to day 14 of culture, as seen in the empirically defined marker analysis.

Interestingly, the considerably high TEER values, functional barrier properties, and the polarized monolayer morphology demonstrated by “Arlo” seem to contradict a proliferating AT‐2‐like character. Primary human proliferating AT‐2 cells, which were cocultured together with fibroblast feeder cells in addition to a treatment with the Rho kinase inhibitor Y‐27632 in order to maintain active proliferation programs in vitro, only demonstrated TEER values of ≈450 Ω cm^2^ when cultured under ALI conditions.^[^ ^30^ ^]^

In contrast, TEER values of ≈3000 Ω cm^2^ as demonstrated by “Arlo” would be associated with an AT‐1‐like in vitro performance, as obtained by our lab for hAEpCs in the past and also demonstrated by one hAEpCs isolation in the current study.^[^ ^29^ ^]^ But neither the cuboidal morphology of “Arlo” cells nor the gene signatures seem to support an AT‐1‐like phenotype. In this context, these new findings strongly challenge our previous assumption that the polyclonal hAELVi cell line is an AT‐1‐like cell line. Others also discussed the lack of strong evidence concerning an AT‐1‐like phenotype, besides CAV1 expression and elevated TEER values.^[^ ^20^ ^]^

The markers CLCA2, DAPL1, LY6D, and TUBB3 that were either defined in the empirical or consensus based marker sets, showed higher abundance in “Arlo” in comparison to hAEpCs. These markers were not only part of the transcriptomic gene signatures representative for basal cells, club cells of nasal origin as well as suprabasal cells but have also been shown to be upregulated in lung cancer patients.^[^ ^87^, ^88^, ^89^, ^90^ ^]^ As already mentioned in the results section, the gene product of CLCA2, the Chloride channel accessory 2 protein, is known to modulate calcium‐activated chloride channel currents and could thus have an influence on the elevated PD values observed for “Arlo.”^[^ ^64^ ^]^ However, the elevated expression of CLCA2 was also shown to be an important characteristic of epithelial differentiation and loss of CLCA2 was discussed to promote breast cancer metastasis.^[^ ^91^ ^]^

Within this context, the cell line NCI‐H441 that was generated from a male patient with a papillary adenocarcinoma also demonstrated mixed characteristics of club cell‐like and AT‐2‐like cells.^[^ ^92^ ^]^ While the presence of SFTPA and SFTPB was detected in these early studies, also the AT‐1 relevant marker RAGE has been reported to be expressed by NCI‐H441 in later studies.^[^ ^93^ ^]^ Similar to the polyclonal hAELVi cell line, also for NCI‐H441 no clonal selection was performed, which a study by Neuhaus et al. supported by identifying two distinct subpopulations of the NCI‐H441 cell line. In one subpopulation the expression of the AT‐1 related markers CAV1 as well as RAGE and in the other population the AT‐2 related marker SFTPB could be detected.^[^ ^93^ ^]^ A more recent comparison of the polyclonal cell line hAELVi and the cell line NCI‐H441 identified SFTPC expression and presence of lamellar bodies in NCI‐H441.^[^ ^50^ ^]^ Although the cell lines NCI‐H441 and the polyclonal hAELVi cell line have been suggested as promising models for the alveolar epithelium especially in the context of biopharmaceutical experiments, both share similar drawbacks in terms of reproducible barrier properties reported in literature. TEER value development of NCI‐H441 seems to strongly depend on the seeded cell density and the addition of dexamethasone to the culture medium.^[^ ^94^ ^]^ TEER values of ≈1500 Ω cm^2^ have been recently described for NCI‐H441 under ALI conditions, while earlier studies demonstrated TEER values of < 300 Ω cm^2^.^[^ ^46^, ^47^ ^]^ In the study by Lochbaum et al., however, TEER values seem to rise until day 5 of culture to values ≈1500 Ω cm^2^ and then seem to decline towards day 7, which strongly restricts the timespan at which experiments could be performed. Another study looked at culture times of 30 d, but only reported maximum TEER values of ≈140 Ω cm^2^ at day 10 of culture for NCI‐H441 cultured under ALI conditions, which also declined rapidly after this maximum.^[^ ^50^ ^]^ In addition, multilayer formation was reported for NCI‐H441 in several studies, further complicating the assessment of TEER development when no quantitative assessment of multilayer formation is performed in parallel.^[^ ^50^, ^95^ ^]^ Similarly, multilayer formation by the polyclonal cell line hAELVi was demonstrated in our study supporting the findings of others.^[^ ^50^, ^96^, ^97^ ^]^

The single‐cell clone “Arlo,” however, demonstrated the formation of a polarized monolayer in a direct comparison with the polyclonal hAELVi cell line under equal culture conditions. The evaluation of the tissue morphology, which is based on quantitative image analysis, demonstrates that the monolayer morphology of “Arlo” is not limited to certain areas within selected sections—it represents the dominant tissue architecture. The tissue architecture of “Arlo” seems to be additionally supported by functional processes relevant to barrier integrity. This can not only be observed by the elevated TEER values together with increased transepithelial PD under LCC and ALI conditions, but also by the expression of genes and synthesis of proteins relevant to barrier formation as well as homeostasis. Among the largely similar gene expression patterns observed for “Arlo” in comparison to hAEpCs a large number of genes coding for the tight junction protein family of claudins can be found.

Especially, the claudins CLDN3, CLDN4, CLDN5, CLDN7, and CLDN18 have been reported to fulfill important functions in the lung epithelium.^[^ ^55^ ^]^ While CLDN5 showed elevated expression in airway epithelial cells, others such as CLDN7 have been shown to be ubiquitously expressed by nearly all epithelial cells in the lung.^[^ ^98^, ^99^ ^]^ CLDN3, CLDN4, and CLDN18 seem to fulfill specific roles in the alveolar epithelium.^[^ ^100^ ^]^ Remarkably, upregulation of CLDN4 and downregulation of CLDN3 through use of an adenoviral vector was reported to increase barrier properties of AT‐1‐like rat cells in vitro.^[^ ^101^ ^]^ All of the patterns mentioned above could be observed in the samples of “Arlo” as well as in the hAEpCs with the exception of CLDN18. The splice variant Claudin 18.1 by the CLDN18 gene is exclusively expressed in the lung and highly expressed in the alveolar epithelium.^[^ ^102^, ^103^ ^]^ While the highest abundance of CLDN18 transcripts could be observed on day 0 for hAEpCs, the abundance declined during the following days of culture. Within samples of “Arlo” transcripts of CLDN18 could not be detected. This finding is again supportive of the proliferating AT‐2 character of “Arlo,” since proliferating AT‐2 cells were highly abundant in CLDN18 double knockout mice.^[^ ^104^ ^]^ Transcripts for CLDN10, which is specifically expressed by club cells, were also absent in “Arlo.”^[^ ^105^ ^]^

Homogenous distribution as well as localization of Occludin and Tight Junction Protein 1/ZO‐1 in the samples of “Arlo” further indicate the presence of intact tight junctional complexes. These findings are also supported by the transcriptomic profiles of the genes coding for these proteins, OCLN and TJP1. Although these tight barrier properties also showed to enable vectorial ion transport in case of “Arlo,” identifying the reasons for the observed increase in transepithelial PD would have exceeded the methods that were available during this study. However, recent reports support our finding of an elevated expression of FXYD3 in alveolar and other respiratory epithelial cells, being highest in AT‐1 cells.^[^ ^106^, ^107^ ^]^ Furthermore, FXYD3 mediated regulation of the sodium/potassium‐transporting ATPase was linked to an increase in sodium absorption in proximal airway epithelia, which also positively stimulated the rate of liquid absorption.^[^ ^108^ ^]^ Again, in the NCI‐H441 cell line, FXYD3 overexpression did not lead to a significant increase in sodium absorption.^[^ ^109^ ^]^ While the quantification of mRNA expression can be used to estimate transporter activity, future studies using “Arlo” would need to include functional assessments of the transport of specific transporter substrates in presence and absence of selective transport inhibitors, to draw conclusions about the elevated transepithelial PD values that are more robust.^[^ ^110^ ^]^

The paracellular transport studies with the low‐permeability marker fluorescein sodium, under the additional influence of EDTA, moreover indicated that the tight junction complexes could be modulated. Modulation of barrier properties could also be seen under the synergistic influence of the inflammatory cytokines TNF‐*α* and INF‐*γ*. The single‐cell clone “Arlo” allowed for the rapid adaption of a protocol by Metz et al., that was established to assess barrier properties of the polyclonal cell line hAELVi under the influence of proinflammatory stimulants.^[^ ^75^ ^]^ We omitted FCS as well as hydrocortisone in our adapted protocol only from the stimulation medium since longer cultivation of the polyclonal cell line hAELVi without FCS and hydrocortisone led to morphological changes.^[^ ^75^ ^]^ Barrier properties of “Arlo” were not negatively affected by the stimulation medium, whereas the synergistic treatment with TNF‐*α* and INF‐*γ* led to a collapse of barrier properties after 48 h treatment. We did, however, not assess, whether the reduction of barrier properties under the synergistic influence of TNF‐*α* and INF‐*γ* was caused by panopsis, as also seen in COVID‐19 patients, or stimulation of other cellular pathways such as NF‐*κ*B.^[^ ^75^, ^76^ ^]^ Further possible applications for “Arlo” beyond the use in classical biopharmaceutical experiments were revealed by the exploration of the RNA‐Seq data for possible protein‐protein interactions using the STRING resource. Interestingly, these data not only supported the findings of interactions among the MHC II surface antigens as also seen in the gene ontology analysis, but also revealed an interaction network that included ACE2. Based on these results we not only verified angiotensin I converting enzyme 2 as well as the serine protease TMPRSS2 on the protein level, but in addition also demonstrated productive infection of “Arlo” with SARS‐CoV‐2. Although we saw productive infection in a total of three different biological replicates, these data need to be interpreted as a proof of principle, since the time points for infection differed with each replicate. Our findings are further supported by a recently published study, which provided evidence that the polyclonal hAELVi cell line could also be used to model productive viral infection, including variants of SARS‐CoV‐2.^[^ ^111^ ^]^ In addition, the study by Mache et al., however, also demonstrated the multilayer formation by the polyclonal hAELVi cell line, which we quantified in the current study. Nonetheless, these findings offer new possibilities to use “Arlo” also as an epithelial model in the context of infection research. Given that ACE2 and other host factors are diversely expressed in the nasal, bronchial and alveolar epithelium by different cell types such as ciliated, club, secretory and alveolar cells, it will be interesting to see to which of these cell types “Arlo” corresponds the most.^[^ ^112^, ^113^ ^]^ Based on our results, the resemblance of an AT‐2‐like phenotype by “Arlo” is most likely and would also fit to in vivo observations showing a higher preference of SARS‐CoV‐2 to infect AT‐2 pneumocytes.^[^ ^114^ ^]^

## 4 Limitations of the Study

As it is with most studies, also the results reported herein should be valued in light of some limitations. We have chosen to perform an untargeted bulk RNA‐seq analysis over single‐cell RNA‐seq analysis of the hAEpCs as well as “Arlo” samples, since bulk RNA‐seq allowed us to obtain a deeper coverage of the total transcriptome and offered a simpler isolation of the RNA representative for all cells grown on the Transwell inserts. While this allowed us to identify many transcriptomic gene signatures that are representative for specific cell types or molecular complexes with confidence in the bulk samples due to a high level of mapped reads, we could not obtain defined clusters of specific cell types that share the same transcriptome. Assigning a specific cell type in the alveolar epithelium, especially given the intricacies of sample preparation and growing cells on Transwell inserts, requires identifying a multidimensional set of appropriate markers. Such future studies could build on advanced spatial proteomics techniques like, e.g., deep visual proteomics to fully unravel the cellular heterogeneity seen in the hAEpCs as well as “Arlo” samples.^[^ ^115^ ^]^

## 5 Conclusion 

In vitro cultures of “Arlo” grown on Transwell inserts at the air interface reliably develop monolayers with functional tight junctions. Low paracellular permeability is the most essential feature of the alveolar epithelium for building a meaningful in vitro model in the context of biopharmaceutical inhalation research. The strong similarity between “Arlo” and primary hAEpCs relating to gene expression involved in the formation of the junctional barriers in the human lung further opens opportunities to use “Arlo” in various other biomedical and biological disciplines. This especially holds true for infection research, where it will be fascinating to study whether viral infection might trigger or even influence antigen presentation by these alveolar epithelial cells.

## 6 Experimental Section

### Cell Culture

The hAELVi polyclonal cell line (CI‐hAELVi; InSCREENex GmbH, INS‐CI‐1015), passages 33 to 36, as well as the single cell clone “Arlo,” passages 1 to 20, were cultured in T25 cm^2^ culture flasks containing 7 mL small airway growth medium (SAGM) Bullet kit (Lonza, CC‐3118) supplemented with 100 U mL^−1^ penicillin, 100 µg mL^−1^ streptomycin (15140122) and 1% fetal calf serum (FCS) (all Gibco, Thermo Fisher Scientific Inc.), which was exchanged every two to three days. Cells were passaged after 7 d of culture. For that purpose, cells were washed twice with 7 mL PBS‐buffer (Dulbecco's PBS; Sigma‐Aldrich, D8537) detached with 2 mL trypsin‐EDTA 0.05% (Gibco, Thermo Fisher Scientific Inc., 25300‐054) for 8 min., centrifuged at 300 rcf for 4 min. The cell suspension was reconstituted from the pellet in 5 mL SAGM Bullet kit including all supplements and cells were seeded into a new T25 cm^2^ culture flask (0.7 × 10^6^ cells per flask) and/or used for the experiments detailed in the following paragraphs. Before cell seeding, the cell culture flasks have been coated for 1 h at 37 °C by using a 2 mL solution of 1% volume/volume (v/v) fibronectin (human 1 mg mL^−1^; Corning, 356008) and 1% (v/v) collagen type 1 (bovine 3 mg mL^−1^, Sigma‐Aldrich, C4243) in distilled and sterile‐filtered H_2_O. The coating solution was completely aspirated shortly before cell seeding. All solutions were prewarmed to 37 °C before use and cells were maintained at 37 °C in a humidified atmosphere containing 5% CO_2_.

### Single‐Cell Isolation and Clonal Expansion

Polyclonal hAELVi cells have been cultured for 7 d in a T25 cm^2^ cell culture flask containing 7 mL SAGM Bullet kit including all supplements until confluent and were handled in the same manner as they would have been for passaging. The only exception was that the cell suspension was reconstituted in HBSS and adjusted to a concentration of 0.7 × 10^6^ cells per mL after the centrifugation step. Mean cell diameter as well as cell diameter distribution of the single cell suspension were measured using a cell counter and analyzer (CASY; OMNI Life Science). A volume of 500 µL of the single‐cell suspension was transferred to a dispensing cartridge of the single‐cell printer (c.sight; Cytena). To collect the printed single cells, a 96‐well cell culture plate was precoated for 1 h with 50 µL per well of the coating solution from Section 1.1. The inner wells (60 wells) were then supplemented with 50 µL SAGM Bullet kit including all supplements and the outer wells (36 wells) were filled with 50 µL distilled and sterile‐filtered H_2_O, to protect the single cell cultures from temperature changes as well as evaporation effects within the 96‐well plate. In addition, the prepared 96‐well plate was placed at 37 °C in a humidified atmosphere containing 5% CO_2_ until the printing procedure started. Single‐cell printing as well as culturing of the single cells was performed according to the manufacturer's instructions with the parameters depicted in Figure 1B (Number of cells per well: 1; Cell diameter [µm]: 10–25; Cell roundness [a.u.]: 0.7–1), after performing a droplet quality control.^[^ ^116^ ^]^ The inner 60 wells of the 96‐well plate containing the printed single cells, whose mono‐clonality was confirmed by the image‐based algorithm, were fed with 8 µL SAGM Bullet kit including all supplements 48 h after the single cell printing without aspirating any medium from the culture wells every second day until bigger colonies of dividing cells (10–20 cells) could be observed. A volume of 8 µL of distilled and sterile‐filtered H_2_O was added to the outer 36 wells. The cell culture medium of wells that contained colonies of dividing cells, was then aspirated every second day and replenished with fresh 200 µL SAGM Bullet kit including all supplements in case of the inner wells or 200 µL of distilled and sterile‐filtered H_2_O in case of the outer wells until cells reached confluency. As exemplarily shown in Figure 1A for “Arlo,” monoclonal cultures were then serially transferred to bigger plate formats once they reached confluency. When cell numbers reached > 0.3 × 10^6^ cells per well they were transferred to a T25 cm^2^ culture flask. Cells from the single‐cell clone “Arlo” were defined as passage 1, after they have been cultured for 7 d in a T25 cm^2^ culture flask for the first time. Passage 1 as well as the consecutive passages were cryo‐preserved for cell banking, where 1 × 10^6^ cells per well were cryopreserved in cryo storage tubes in a cryopreservation medium (35% SAGM Bullet kit including all supplements, 35% Dulbecco's modified Eagle medium/nutrient mixture F‐12 (DMEM/F12; Gibco, Thermo Fisher Scientific Inc., 11320033), 20% FCS as well as 10% dimethyl sulfoxide (DMSO); all volume/volume).

### Isolation of Primary Alveolar Epithelial Cells

Primary alveolar epithelial cells (hAEpCs) have been isolated from lung tissue which had been resected at the SHG clinics Völklingen, Germany according to the procedure described in Daum et al.^[^ ^48^ ^]^ The local ethics committee of the state of Saarland, Germany, permitted the use of the patient material for the biomedical research performed in this study on 21 May 2019 under the sign 113/19. The committee has reviewed the patient consent forms in this process as well.

Briefly, the lung tissue was chopped into 5 µm wide smaller pieces using a tissue chopper (McIlwain Tissue Chopper, Plano GmbH). These pieces were collected in a 50 mL Falcon tube containing 30 mL balanced salt solution (137 × 10^−3^ m NaCl, 5 × 10^−3^ m KCl 0.7 × 10^−3^ m, Na_2_HPO_4_ 10 × 10^−3^ m, HEPES, 5.5 × 10^−3^ m glucose, adjusted to pH 7.4) and washed three times by using a 100 µm pore‐sized cell strainer (Greiner, 542000) to recover the tissue remnants during each washing step. The tissue remnants were then enzymatically digested to generate a cell suspension, by using a combination of 1.5 mL trypsin (1 × 10^6^ BAEE units mL^−1^; Sigma‐Aldrich, T8003) and 300 µL elastase (10 mg mL^−1^, Worthington, LS002279) for 40 min at 37 °C. After this incubation time, the cell suspension was serially washed again by using a 100 µm pore‐sized cell strainer first, followed by an washing step through a 50 µm pore‐sized cell strainer (Greiner, 542040). By incubating the cell suspension in cell culture petri dishes for 90 min at 37 °C, attaching immune cells as well as erythrocytes were excluded from the suspension. The cell suspension was then further purified using a Percoll gradient (Sigma‐Aldrich, P1644) followed by a positive antibody selection for CD326 (EpCam) positive cells using a LS column (Micro beads; MACS column; Miltenyi Biotec, 130‐061‐101 (beads) or 130‐042‐401 (column)). The cell suspension of purified CD326‐positive cells was recovered in 5 mL SAGM Bullet kit including all supplements and either seeded on Transwell inserts as described in the following paragraph, or used for isolation of RNA.

### Transwell Experiments

Before cell seeding, Transwell inserts were coated for 1 h at 37 °C by adding a 100 µL solution of 1% (v/v) fibronectin (human 1 mg mL^−1^; Corning, 356008) and 1% (v/v) collagen type 1 (bovine 3 mg mL^−1^, Sigma‐Aldrich, C4243) in distilled and sterile‐filtered H_2_O per Transwell. The coating solution was completely aspirated shortly before cell seeding.

0.33 × 10^5^ cells of the polyclonal cell line hAELVi as well as the single cell clone “Arlo” or 1 × 10^5^ cells of the hAEpCs were seeded in 200 µL SAGM Bullet kit including all supplements per apical compartment of a Transwell insert (0.33 cm^2^; 400 nm pore size; Corning, 3470) (1 × 10^5^ cells cm^−^^2^, cell lines; 3 × 10^5^ cells cm^−^^2^, hAEpCs). The basolateral compartment was supplemented with 800 µL SAGM Bullet kit including all supplements. Every 2 to 3 d used medium was aspirated from the basolateral compartment first and then from the apical compartment. In the case of liquid‐covered conditions (LCC) SAGM Bullet kit including all supplements was supplemented first in the apical compartment (200 µL) followed by the basolateral compartment (800 µL). Air–liquid interface (ALI) conditions were established on day 3 of culture by aspirating the medium from the apical compartment and supplementation of 400 µL SAGM Bullet kit including all supplements in the basolateral compartment.

### Electrophysiological Measurements: Transepithelial Electrical Resistance (TEER) Measurements

TEER measurements were conducted for all cells cultivated under LCC as well as ALI conditions on Transwell inserts, 2 h after LCC conditions have been restored by medium exchange. TEER was measured with a chopstick electrode connected to a Volt‐Ohm‐meter (STX2 and EVOM 2; World Precision instruments) according to the manufacturer's instructions. During the time of the measurement, the Transwell plate was placed on a heating plate (37 °C). Ohmic resistance values (Ω) were corrected for the area of the Transwell insert (0.33 cm^2^) as well as a blank (when no blank Transwell insert was available in the experiment, a value of 100 Ω was used by default) and reported as Ω cm^2^. If not described differently, medium exchange was performed after each TEER measurement for cells cultured under LCC as well as ALI conditions.

### Epithelial Potential Difference (PD) Measurements

The STX2 electrode was immersed in a solution of 0.15 (m) KCl in distilled and sterile‐filtered H_2_O for 2 h before every voltage measurement while connected to a switched off EVOM 2 according to the manufacturer's instructions. This procedure should assure voltage stability and a low inter‐electrode potential difference. Voltage values (mV) for all cells cultivated under LCC as well as ALI conditions on Transwell inserts, were measured 2 h after LCC conditions have been restored by medium exchange and were corrected for the related blank. TEER measurements were always performed after PD measurements.

### Transport Experiments

Before each transport experiment, TEER of the polyclonal hAELVi and the single cell clone “Arlo” cell lines cultured until day 14 under LCC were measured to determine barrier integrity before the experiment (before). Cells were washed once with pre‐warmed Hanks’ balanced salt solution (HBSS) with CaCl_2_ as well as MgCl_2_ (HBSS (1×); Gibco, Thermo Fisher Scientific Inc., 14025050) and then equilibrated in HBSS (200 µL apical; 800 µL basolateral) for 1 h (1 h after switch) in case of the control group. To disrupt the integrity of tight junctions, the treatment group was equilibrated for 1 h (1 h after switch) in HBSS containing 2,2′,2″,2′″‐(ethane‐1,2‐diyldinitrilo)tetraacetic acid (EDTA) (16 × 10^−3^ m). After measuring TEER, HBSS was aspirated from both, the apical and basolateral compartment. 200 µL fluorescein sodium solution (2.5 µg mL^−1^ in HBSS) was added apically (donor) and 800 µL HBSS was added to the basolateral compartment (acceptor). From the same solutions, 200 µL each was transferred into a 96‐well plate to determine the starting concentrations for each compartment. All steps were performed on a heating plate at 37 °C. Afterward, the Transwell plates were placed on a MTS orbital shaker (150 rpm; IKA, Germany) in the incubator and 200 µL samples were taken every 1 h for a total of 5 h, from the basolateral compartment only. 200 µL sampled at time points were immediately replenished with 200 µL prewarmed HBSS. TEER was measured 30 min after the last sample was taken (after 6 h), 200 µL from the apical as well as the basolateral compartment were sampled to determine the end concentrations and all samples were measured with a plate reader in a 96‐well plate at 485 nm excitation and 530 nm emission wavelength. The concentration of fluorescein sodium in each sample was calculated using a calibration curve of defined concentrations of fluorescein sodium in HBSS.

### Calculation of the Apparent Permeability Coefficient (*P*_app_)

Sink conditions, where drug concentration in the receiver compartment should not exceed 10% of the drug concentration added to the donor compartment at the start of the experiment, were ensured during the assay. The flux of fluorescein sodium (J) [ng cm^−^^2^ s^−1^] was calculated by dividing the slope from the linear section of the cumulative concentration–time curve (60– 180 min) of the transported fluorescein sodium solution and divided by the area (*A*) [cm^2^] of the growth support. To obtain the apparent permeability (*P*app) [cm s^−1^] the following equation was applied, where the initial concentration in the donor compartment at the beginning of the experiment is defined as *c*_0_ [ng cm^−^^3^]:

(1) $$P\mathrm{app}=\frac{J}{C_{0}}$$

### Growth Curve

For the growth curve experiments, 3000 cells per well of the polyclonal hAELVi cell line and the single cell clone “Arlo” were seeded on 24‐well cell culture plates on day 0 which have been precoated with 400 µL coating solution for 1 h at 37 °C as described above. Every day the cells from 3 wells per cell line were collected. This was done by washing each well first with 300 µL PBS, followed by cell detachment with 200 µL trypsin–EDTA 0.05% and the addition of 400 µL PBS containing 1% FCS (v/v) to stop the trypsin reaction after the cells had been detached. The cell suspension was then transferred to a 2 mL reaction tube and centrifuged at 300 rcf for 4 min, before the cell suspension was reconstituted from the pellet in 400 µL PBS containing 1% FCS (v/v). The cells were counted with a CASY cell counter (capillary size: 150 µm, size scale 0–50 µm, sample volume 10 × 400 µL) by transferring 200 µL of the cell suspension to 10 mL of CASY tone (dilution factor: 51). Total cell number was reported as total cells cm^−^^2^ after correcting for the dilution as well as the growth area of the culture well (0.95 cm^2^).

### Inflammation Assay

“Arlo” cells have been cultured for 10 days under LCC conditions on Transwell inserts in SAGM Bullet kit including all supplements as described in Section 1.1.3, as an adapted version of the protocol from Metz et al.^[^ ^75^ ^]^ Last medium exchange with SAGM Bullet kit including all supplements was performed on day 9 of culture. On day 10 of culture TEER values (prestimulation values) were determined before SAGM Bullet kit including all supplements was exchanged either against stimulation medium or stimulation medium supplemented with tumor necrosis factor alpha (TNF *α*) (human 25 ng mL^−1^; Sigma‐Aldrich, H8916‐10UG) and interferon gamma (INF *γ*) (human 30 ng mL^−1^; Sigma‐Aldrich, I17001‐100UG). The stimulation medium contains all the components of the SAGM Bullet kit, but hydrocortisone as well as FCS were omitted. “Arlo” cells were incubated in stimulation medium or stimulation medium supplemented with TNF *α* and INF *γ* for 48 h without exchanging the cell culture medium. TEER measurements were performed after 24 and 48 h and normalized to the prestimulation values.

### Imaging: Confocal Laser Scanning Microscopy: Immunofluorescence Staining

If not stated otherwise, cells prepared for immunofluorescence staining were cultured on Transwell inserts for 7 or 14 d under ALI conditions. First, the remaining medium was aspirated first from basolateral and then from the apical compartment of the Transwells, before the Transwells were washed starting with the apical compartment followed by the basolateral compartment with pre‐warmed PBS (apical: 200 µL, basolateral: 800 µL). Fixation was performed with 200 µL of 4% paraformaldehyde (in PBS) for 10 min at room temperature (RT) from apical only. Permeabilization as well as blocking of unspecific epitopes was conducted with blocking buffer (1% BSA (bovine serum albumin heat shock fraction; Sigma‐Aldrich, A9647‐50G), 0.05% Saponin (Saponin Quillaja sp.; Sigma‐Aldrich, S4521‐10G) in PBS (weight/weight/volume)) for 1 h at RT. Primary antibodies against tight junction proteins Occludin (monoclonal antibody, Thermo Fisher Scientific, Cat# 33‐1500) or ZO‐1 (monoclonal antibody, BD Biosciences, Cat# 610966) were both diluted (1:200 (v/v)) in blocking buffer and incubated for 12 h at 4 °C in cases where antibody stainings were performed. The related secondary antibody conjugated to Alexa 633 (polyclonal antibody, Thermo Fisher Scientific, Cat# A‐21050) (1:2000 (v/v) in blocking buffer) was incubated for 1 h at RT. F‐Actin staining via rhodamine phalloidin (1:200 (v/v) in blocking buffer) (Invitrogen, R415) was either conducted instead of the antibody staining, or conducted after the third washing step of the secondary antibody, for 45 min at RT. Nuclei were stained with DAPI (1 µg mL^−1^ in PBS (v/v)) for 30 min at RT. A volume of 200 µL was used for all the steps mentioned above and Transwells were washed in between every step with PBS at RT three times for 10 min. After the staining procedure, the membrane of the Transwell was carefully detached from the plastic holder using a forceps, by slowly inserting a scalpel in the outer boundary of the membrane. Briefly, the membrane was cut from the plastic holder in a circular manner using a scalpel, mounted on a microscope slide (Superfrost; Menzel, AAAA000080##32E) and embedded with fluorescence mounting medium (DAKO, S3023). Samples were always kept moist by careful addition of PBS during the cutting and mounting procedure.

### Image Acquisition

Representative micrographs of cells stained by indirect fluorescence were acquired as z‐stacks with an inverted confocal laser scanning microscope (CLSM; Leica, TCS SP8). The microscope was equipped with a 25× (Fluotar VISIR 25×/0.95) as well as a 60× water immersion objective (60× HC PL APO CS2 63×/1.20). The Resolution was set to 1024×1024 pixels, the scan speed to 200 Hz and the z‐step size to 356 nm. Z‐stacks were acquired as xy‐scans moving in z‐direction. Fluorescence of DAPI representing the cell nuclei was detected with the emission filter set to 463–494 nm excited by a diode laser at 405 nm. Fluorescence of rhodamine coupled to phalloidin representing the cellular F‐Actin network was detected with the emission filter set to 580–609 nm excited by a laser line at 561 nm. ZO‐1 or Occludin signals representative for the tight junctional networks were detected with the emission filter set to 650–681 nm excited by a laser line at 633 nm. Hybrid detectors (HyD) of the microscope were set to “photon‐counting mode” with a line accumulation set to a value of 3, in order to improve the sequential quantification of the intensity signal, for the ZO‐1 as well as Occludin signal. Orthogonal optical sections were either computationally reconstructed from z‐stack images using FIJI/Image or acquired directly on the microscope using the xzy‐scan mode of the Leica SP8. Maximum intensity projections of z‐stacks were created with the same settings for all images with FIJI/Image J^[^ ^117^ ^]^ and further equally processed using the BIOP Channel tools plugin (https://c4science.ch/w/bioimaging_and_optics_platform_biop/image‐processing/imagej_tools/ijab‐biop_channel_tools/).

### Computational Image Analysis

Z‐stacks which were used for the computational image analysis, were all acquired with the 25× water immersion objective in a resolution of 1024 × 1024 pixels, a thickness of 60 µm, a z‐step size of 1 µm and a scan speed of 200 Hz from the center of each Transwell membrane. HyD detectors of the microscope were set to “photon‐counting mode” with a line accumulation set to a value of 4, in order to improve the sequential quantification of the intensity signal. Fluorescence of DAPI representing the cell nuclei was detected with the emission filter set to 420–451 nm excited by a diode laser at 405 nm. Fluorescence of rhodamine coupled to phalloidin representing the cellular F‐Actin network was detected with the emission filter set to 581–607 nm excited by a laser line at 561 nm. 3D surface creation with Imaris (Bitplane AG) generated individual 3D‐objects based on the fluorescence intensity, in our case the DAPI signal of each nucleus, and allowed to extract individual statistics for each of these 3D‐objects (in our case the 3D‐position of the center of each nucleus in a z‐stack). For the 3D‐surface creation, a single image from each group (polyclonal cell line hAELVi ALI on day 14 vs single cell clone “Arlo” ALI on day 14) was used to define the parameters for the computational algorithm. All other samples were analyzed using these initially set parameters with the Imaris batch analysis, to reduce observer bias. Due to an error during the automated image‐acquisition, the only parameter that differed between the two groups (polyclonal cell line hAELVi vs single cell clone “Arlo”) was the digital zoom (polyclonal cell line hAELVi: 1; single cell clone “Arlo”: 1.28). This difference had no influence on the relative nuclei position and was taken into account during the creation of the 3D surface by separating the two groups. Position statistics were exported and analyzed using GraphPad Prism 9.3.1. software.

### Histological Micrographs

Samples of the polyclonal cell line hAELVi or the single cell clone “Arlo” were cultured under ALI conditions until day 7 or day 14 and then fixated as described in Section 1.2.1. They were covered afterward with HBSS (apical: 200 µL, basolateral: 800 µL). Samples were processed according to the following protocol of the supplier and stained with hematoxylin and eosin.^[^ ^118^ ^]^

### Bulk RNA‐Sequencing and Analysis: RNA Library Preparation

hAEpCs and cells “Arlo” were prepared for RNA‐isolation on day 0 (hAEpCs: 300.000 cells, directly after the isolation; “Arlo” 300.000 cells, directly after passaging) or on day 7 as well as day 14 from cells grown under ALI conditions on Transwell inserts. Cells were washed twice with 200 µL prewarmed HBSS and RNA was subsequently isolated by using the Direct‐zol RNA Microprep Kit (Zymo Research, R2062) following the manufacturer´s instructions. RNA from hAEpCs on day 0 and day 7 in 100 µL and on day 14 in 50 µL TRI‐reagent (Zymo Research, R2050‐1‐50) for 5 min at RT and directly put on ice. To isolate RNA from the cells of “Arlo” on day 0 100 µL, on day 7 150 µL and on day 14 200 µL TRI‐reagent was used to compensate for the higher cell numbers of “Arlo.” A modified SmartSeq 2 protocol was followed where total RNA between 2 and 100 ng was used as input for reverse transcription.

RNA was primed by adding 1 × 10^−6^ m Oligo‐dT Primer (final conc.; 5´AAGCAGTGGTATCAACGCAGAGTACTTTTTTTTTTTTTTTTTTTTTTTTTTTTTTVN; V = A/C/G, N = any base), 1 × 10^−3^ m dNTPs (final conc.) followed by a denaturation step at 72 °C for 3 min and immediately cooling on ice. Reverse transcription was performed in a 10 µL volume reaction by using 0.5 µL Superscript II RT (200 U µL^−1^, Thermo Fisher Scientific, 18064022), 0.4 µL RNAse inhibitor (40 U µL^−1^, Promega, N2515), 5 × 10^−3^ m DTT, 1 m Betaine, 6 × 10^−3^ m MgCl_2_ and 1 × 10^−6^ m TSO (B‐AAGCAGTGGTATCAACGCAGAGTACAT997, B = 5’ biotin, 7 = LNA g, 9 = RNA‐G) under the following incubation conditions: 42 °C for 90 min, 10 × cycling of 50 °C for 2 min and 42 °C for 2 min, finalized by 70 °C for 15 min.

The preamplification of the cDNA was carried out by utilizing the KAPA HiFi HotStar Ready Mix (Roche, KK2601) and 0.1 × 10^−6^ m of the IS PCR primers (5´AAGCAGTGGTATCAACGCAGAGT) in a 25 µL volume reaction under the following PCR conditions: 98 °C for 3 min, 10–12× cycling of 98 °C for 20 s, 67 °C for 15 s, 72 °C for 6 min and a final elongation at 72 °C for 5 min. The cDNA was purified by the use of 0.8× Agencourt AMPure XP Beads (Beckman Coulter, A63881), quantified by the help of Qubit dsDNA HS Assay Kit (Thermo Fisher Scientific, Q32851) and the cDNA integrity was examined via the analysis of the fragment size distribution by using the Agilent 2100 Bioanalyzer (High Sensitivity DNA Analysis Kit, Agilent, 5067‐4626).

The libraries were prepared by applying a tagmentation‐based approach using the Nextera DNA Library Preparation Kit (Illumina, FC‐131‐1024). 8 ng of each cDNA were tagmented for 10 min at 55 °C by the use of 1 µL of the Tagment DNA Enzyme 1 in a 20 µL reaction pursued immediately by the purification of the tagmented fragments by the use of the MinElute PCR Purification Kit (Qiagen, 28004) following the manufacturer's instructions. The amplification of the libraries was performed in a 30 µL reaction using the NEBNext High‐Fidelity 2X PCR Master Mix (New England Biolabs, M0541S) and 0.33 × 10^−6^ m indexed adapters (5′AATGATACGGCGACCACCGAGATCTACAC[i5]TCGTCGGCAGCGTC and 5″CAAGCAGAAGACGGCATACGAGAT[i7]GTCTCGTGGGCTCGG; Illumina). 8 PCR cycles were done of the following PCR program: 75 °C 5 min, 98 °C 10 s, cycling of 98 °C 30 s, 63 °C 30 s and 7 2 °C 1 min, finalized by a long elongation at 72 °C for 7 min. The libraries were purified by utilizing 0.9 × Agencourt AMPure XP Beads, the DNA concentrations were quantified by the help of the Qubit dsDNA HS Assay Kit and the size distribution of the amplified fragments was examined by the use of the Agilent 2100 Bioanalyzer (High Sensitivity DNA Analysis Kit). The libraries were sequenced on the Illumina HiSeq 2500 by using the 100 bp single read sequencing mode.

### m‐RNA Sequencing Data Processing

Adapter sequences of FastQ format RNA‐seq reads were removed and trimmed of low‐quality ends (phred score = 20) by the use of Trim Galore! (version 0.4.2).^[^ ^119^ ^]^ The reads were aligned to the hg38 reference genome (Genbank: GCA_000001405.15) by using grape‐nf (version 433e7621f6),^[^ ^120^ ^]^ which combines STAR (version 2.4.0j)^[^ ^121^ ^]^ for the alignment and RSEM (version 1.2.21)^[^ ^122^ ^]^ for the read assignment. Differential analysis was carried out by the utilization of the software R and the included R‐package EdgeR (version 3.20.9).^[^ ^123^ ^]^ Differentially expressed genes were defined by a maximal *p*‐value of 0.01, a FDR ≤ 0.01 and a minimal log fold‐change of │1│.

### Utilization of the STRING Database

The differentially expressed genes resulting from the pairwise differential analyses by the use of EdgeR were loaded into the STRING database to detect possible protein–protein interactions.^[^ ^69^, ^70^ ^]^ In order to do so the minimum required interaction score was set to the highest confidence (0.9).

### Gene Ontology Annotations Using Gene Ontology

Gene ontology annotations of the identified differentially expressed genes resulting from the pairwise comparisons were done by the use of GENEONTOLOGY.^[^ ^72^, ^73^ ^]^ The differentially expressed genes were restricted to those genes following a linear regression along the cultivation period (d0 to d14) derived by calculation using R. These genes (e.g., higher expressed in d0 and defined by a loss of transcription along the cultivation period) were loaded into GENEONTOLOGY and the resulting GO terms were filtered by a false discovery rate (FDR) of ≤ 0.01 as well as ranked by the enrichment score (provided by GENEONTOLOGY).

### SARS‐CoV‐2 Infection Experiments

The isolates SARS‐CoV‐2/1/Human/2020/Frankfurt (SARS‐CoV‐2/FFM1), SARS‐CoV‐2/7/Human/2020/Frankfurt (SARS‐CoV‐2/FFM7), as well as the variants Alpha (B.1.1.7), Beta (B.1.351) and Zeta (P.) were isolated and produced in Caco‐2‐F03 cells as previously described.^[^ ^124^, ^125^, ^126^ ^]^

“Arlo” cells were cultured on Transwell inserts for 7 or 14 d under ALI conditions before infection with SARS‐CoV‐2/FFM7 was initiated. For the infection studies comprising the variants SARS‐CoV‐2/FFM1, SARS‐CoV‐2/FFM7, Alpha (B.1.1.7), Beta (B.1.351) and Zeta (P.2), “Arlo” cells were cultured on Transwell inserts for 12 d under ALI conditions. “Arlo” cells were infected at a multiplicity of infection (MOI) of 1 from the apical side. After 2 hours, the inoculum was removed by aspiration and cells were washed three times with PBS.

### Quantitative Reverse Transcription PCR (RT‐qPCR) Analysis

On d0 after the third wash with PBS, as well as on days 1, 3, and 5 postinfection, a 30 min wash with PBS was performed from apical to harvest SARS‐CoV‐2 RNA for the RT‐qPCR analysis, as previously described.^[^ ^127^, ^128^ ^]^ SARS‐CoV‐2 RNA was isolated from the apical wash samples via AVL lysis buffer (Qiagen, 19073) and the QIAamp Viral RNA Kit (Qiagen, 52904) following the instructions of the manufacturer. The RNA yield was quantified via absorbance measurement on a Genesys 10S UV‐vis spectrophotometer (Thermo Fisher Scientific). After that a one‐step RT‐qPCR reaction was performed using the Luna Universal One‐Step RT‐qPCR Kit (New England Biolabs, E3006L) as well as a CFX96 Real‐Time System, C1000 Touch Thermal Cycler (Bio‐Rad). Primers, adapted from,^[^ ^129^ ^]^ which target the open reading frame for RNA‐dependent RNA polymerase (RdRp): RdRP_SARSr‐F2 (GTG ARA TGG TCA TGT GTG GCG G) and RdRP_SARSr‐R1 (CAR ATG TTA AAS ACA CTA TTA GCA TA) were used in a concentration of 0.4 × 10^−6^ m per reaction.

### Western Blot Analysis

On days 0, 1, 3, and 5 postinfection “Arlo” cells were lysed for Western blot analysis using Triton‐X‐100 sample buffer, as described previously.^[^ ^130^ ^]^ Proteins were separated by SDS‐PAGE. Specific antibodies against SARS‐CoV‐2 N (1:1000 dilution, SARS‐CoV‐2 Nucleocapsid Antibody, Rabbit monoclonal antibody (Mab), #40143‐R019, Sino Biological), ACE2 (1:500 dilution, Anti‐ACE2 antibody, #ab15348, Abcam), TMPRSS2 (1:1000 dilution, Recombinant Anti‐TMPRSS2 antibody [EPR3861], #ab92323, Abcam), and GAPDH (1:1000 dilution, Anti‐G3PDH Human Polyclonal Antibody, #2275‐PC‐100, Trevigen) allowed antigen detection. Protein bands were made visible by laser‐induced fluorescence using an infrared scanner for protein quantification (Odyssey, Li‐Cor Biosciences).

## Conflict of Interest

C.‐M.L., N.S.‐D., and P.C. are the creators of the cell line “Arlo”. A manufacture and distribution license for the cell line “Arlo” was granted to InSCREENeX GmbH, Germany by the Helmholtz Centre for Infection Research (Helmholtz‐Zentrum für Infektionsforschung GmbH) (HZI), Germany. The authors declare that the research was conducted in the absence of any commercial or financial relationships that could be construed as a potential conflict of interest.

## Supporting information

Supporting Information

## Data Availability

The data that support the findings of this study are available on request from the corresponding author. The RNA‐Sequencing dataset is also available at the research data archive RADAR (number 893). These data are not publicly available due to privacy or ethical restrictions.
