# Supplementary material for: A Monoclonal Human Alveolar Epithelial Cell Line (“Arlo”) with Pronounced Barrier Function for Studying Drug Permeability and Viral Infections
Source: Adv Sci (Weinh). 2023 Feb 7;10(8):2207301. doi: 10.1002/advs.202207301 (PMC10015904; doi:10.1002/advs.202207301)
Supplement: Supplementary file 1 — Supporting Information [file ADVS-10-2207301-s001.pdf]

ADVANCED SCIENCE
Open Access

Supporting Information

for *Adv. Sci.*, DOI 10.1002/advs.202207301

A Monoclonal Human Alveolar Epithelial Cell Line ("Arlo") with Pronounced Barrier Function for Studying Drug Permeability and Viral Infections

Patrick Carius, Annemarie Jungmann, Marco Bechtel, Alexander Grißmer, Annette Boese, Gilles Gasparoni, Abdulrahman Salhab, Ralf Seipelt, Klaus Urbschat, Clémentine Richter, Carola Meier, Denisa Bojkova, Jindrich Cinatl, Jörn Walter, Nicole Schneider-Daum and Claus-Michael Lehr**

Supporting Information

A monoclonal human alveolar epithelial cell line ("Arlo") with pronounced barrier function for studying drug permeability and viral infections

Patrick Carius1,2, Annemarie Jungmann3, Marco Bechtel4, Alexander Grißmer5, Annette Boese1, Gilles Gasparoni3, Abdulrahman Salhab3, Ralf Seipelt6, Klaus Urbschat6, Clémentine Richter1,2, Carola Meier5, Denisa Bojkova4, Jindrich Cinatl4, Jörn Walter3, Nicole Schneider-Daum1* and Claus-Michael Lehr1,2*

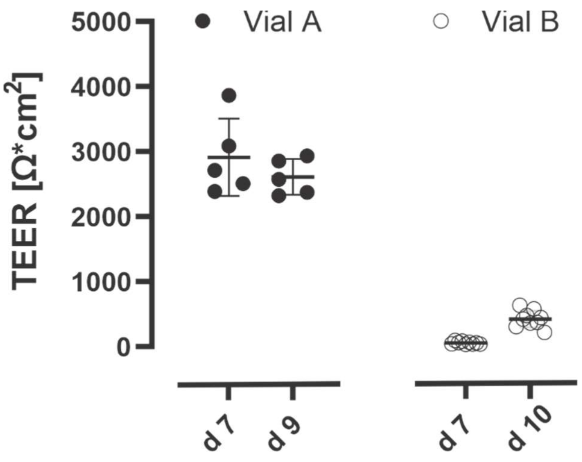

Supplementary Figure 1 Impaired TEER development of the polyclonal hAELVi cell line. Two vials ,Vial A and Vial B, of the polyclonal hAELVi cell line were thawed and passaged once, before cells were seeded on Transwell® inserts as described in the Experimental section. Shown are mean values from TEER measurements from 3 individual experiments with technical replicates indicated in the plot.

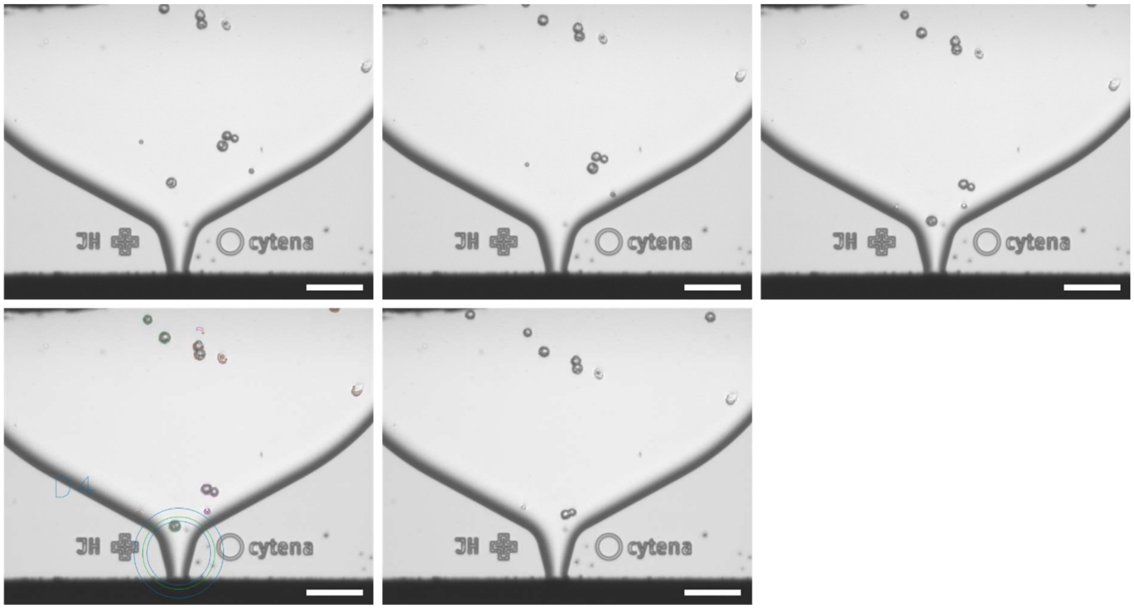

Supplementary Figure 2 Single cell printing sequence. The three pictures in the upper panel display the travel of cells to the printing nozzle before printing of the single cell that was used to generate hAELVI “Arlo“ in well D4 of a 96-well culture plate. The first picture of the bottom panel shows the event of single cell printing (detailed in Figure 1) and the last picture in the bottom panel shows the remaining cells after the printing of the single cell. Scale bar: 100 µm

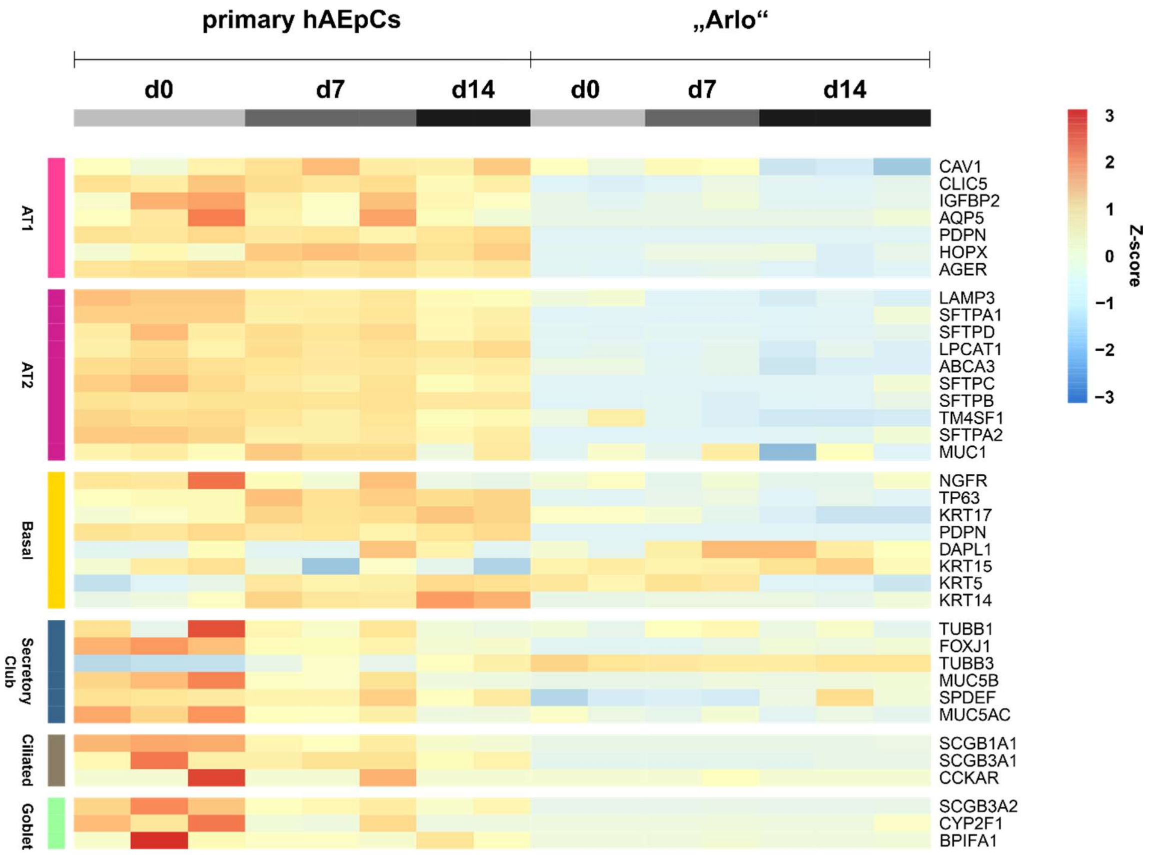

Supplementary Figure 3 Empirical lung epithelial cell marker expressed by hAELVi "Arlo". Cell type specific gene signatures of genes whose expression is representative for specific epithelial cell types within the human lung were derived from [15]. Data represent at least 2 biological replicates of cells cultured under ALI conditions and were derived from bulk RNA-Sequencing.

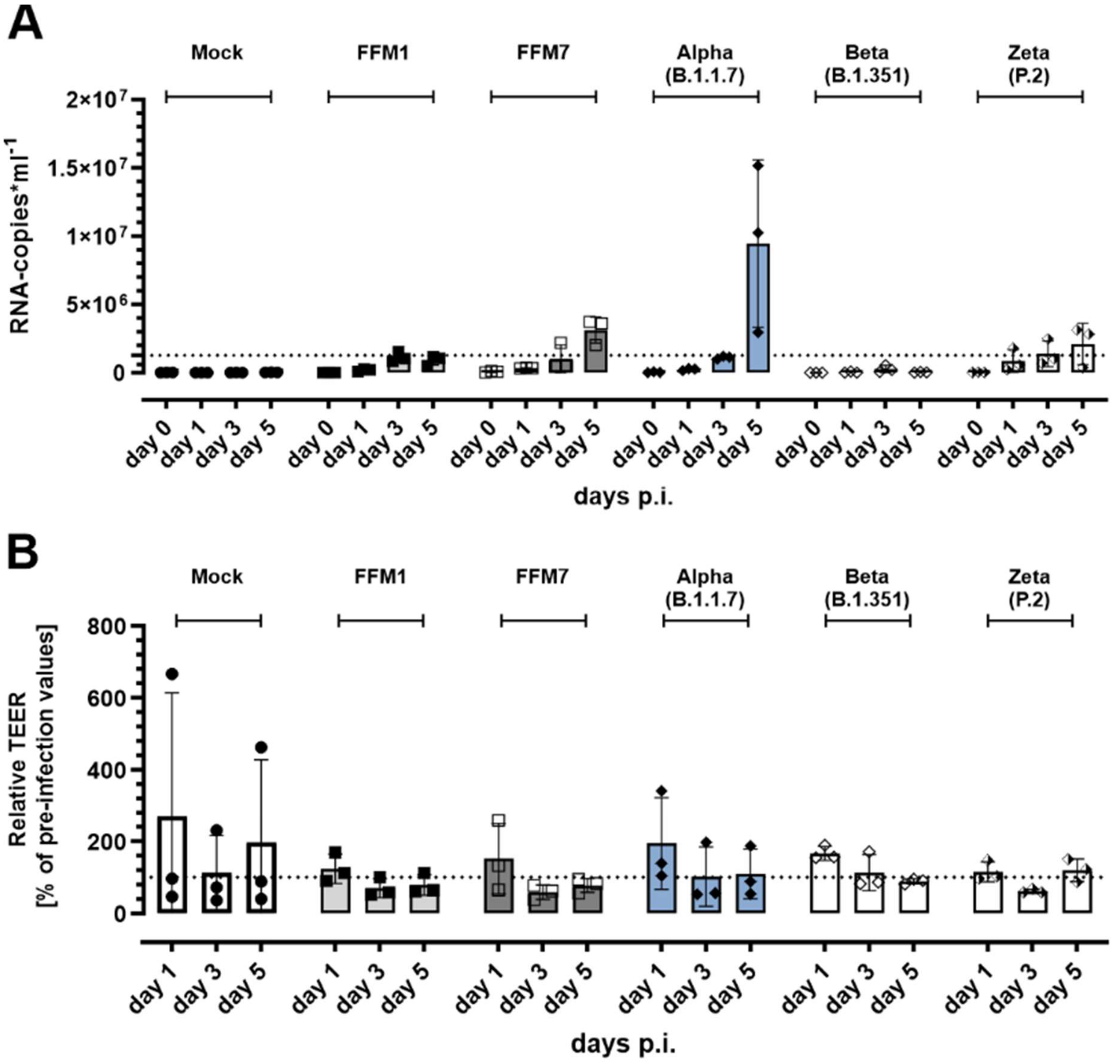

Supplementary Figure 4 SARS-CoV-2 infection of hAELVi "Arlo" with different viral variants. A) Infection studies performed with the single cell clone hAELVi "Arlo" either on day 12 of culture under ALI conditions with SARS-CoV-2 (different variants are indicated) (MOI of 1) or mock (PBS). SARS-CoV-2 RNA copy numbers (RNA-copies*ml-1) derived from qRT-PCR of the RNA-dependent RNA polymerase (RdRp) gene copies present in single apical washes (30 min, PBS) on the given days post-infection (days p.i.). B) Reduction of barrier properties of hAELVi "Arlo" after infection with the different variants. TEER values were normalized to the values before infection (dotted line). Data represent mean ± S.D.; n = 3 for each group from single biological specimen.
